# Uncovering Buffered Pleiotropy: A Genome-Scale Screen for *mel-28* Genetic Interactors in *Caenorhabditis elegans*

**DOI:** 10.1534/g3.113.008532

**Published:** 2013-11-26

**Authors:** Anita G. Fernandez, Emily K. Mis, Allison Lai, Michael Mauro, Angela Quental, Carly Bock, Fabio Piano

**Affiliations:** * Fairfield University Biology Department, Fairfield, Connecticut 06824; † New York University Department of Biology and Center for Genomics and Systems Biology, New York, New York 10003; ‡ New York University, Abu Dhabi, United Arab Emirates

**Keywords:** synthetic sterility, *C. elegans*, gonad, germline, *mel-28*

## Abstract

*mel-28* (*maternal-effect-lethal-28*) encodes a conserved protein required for nuclear envelope function and chromosome segregation in *Caenorhabditis elegans*. Because *mel-28* is a strict maternal-effect lethal gene, its function is required in the early embryo but appears to be dispensable for larval development. We wanted to test the idea that *mel-28* has postembryonic roles that are buffered by the contributions of other genes. To find genes that act coordinately with *mel-28*, we did an RNA interference−based genetic interaction screen using *mel-28* and wild-type larvae. We screened 18,364 clones and identified 65 genes that cause sterility in *mel-28* but not wild-type worms. Some of these genes encode components of the nuclear pore. In addition we identified genes involved in dynein and dynactin function, vesicle transport, and cell-matrix attachments. By screening *mel-28* larvae we have bypassed the requirement for *mel-28* in the embryo, uncovering pleiotropic functions for *mel-28* later in development that are normally provided by other genes. This work contributes toward revealing the gene networks that underlie cellular processes and reveals roles for a maternal-effect lethal gene later in development.

A defining feature of eukaryotes is the nuclear envelope, which separates the DNA within the nucleus from the cytoplasm. The nuclear envelope consists of a double membrane punctuated by nuclear pores, which traverse both membranes of the nuclear envelope and regulate trafficking between the nucleoplasm and the cytoplasm. Nuclear pores are composed of at least 30 nucleoporins, which form several subcomplexes that occur repeatedly within the pore, producing an eightfold rotational symmetry (Stewart 1992; Hoelz *et al.* 2010). In addition to their role in nuclear pore, some nucleoporins play roles in the spindle assembly checkpoint, kinetochore assembly, cytokinesis, regulation of gene expression, and cell migration (Chatel and Fahrenkrog 2011, 2012).

Metazoans have open mitosis, in which the nuclear envelope breaks down completely to allow the spindle apparatus to contact the chromosomes and promote their segregation to opposite poles of the dividing cell. Soon after the onset of anaphase, the nuclear envelope starts to reform around the condensing chromosomes. It has been demonstrated in *Xenopus* extracts and HeLa cells that the binding of MEL-28/ELYS to chromatin is a key early step in the reestablishment of the nuclear envelope (Franz *et al.* 2007; Rasala *et al.* 2008). MEL-28/ELYS then recruits the Nup107-160 complex of the nuclear pore, which in turn recruits other nuclear pore components (Franz *et al.* 2007). Thus, the proper assembly of the nuclear pore requires MEL-28/ELYS. In *C. elegans*, *mel-28* disruption leads to severe nuclear envelope defects (Fernandez and Piano 2006; Galy *et al.* 2006).

In addition to its key roles in the nuclear envelope, *mel-28* is implicated in chromosome segregation. *mel-28* RNA interference (RNAi)-treated animals have abnormally condensed chromatin during early embryogenesis and their chromosomes fail to congress to the metaphase plate, leading to aberrant chromosome segregation (Fernandez and Piano 2006). Some kinetochore components are not recruited to the kinetochore and the mitotic spindle does not form. Knockdown of ELYS in HeLa cells produces cytokinesis defects as well as nuclear envelope defects, and MEL-28/ELYS shuttles between the nuclear envelope and the kinetochore during mitosis is *C. elegans* and in HeLa cells (Fernandez and Piano 2006; Galy *et al.* 2006; Rasala *et al.* 2006).

Because *mel-28* is a gene with crucial functions in both the nuclear envelope and in chromosome segregation, it might be expected to be required in all cells. Consistent with this, the MEL-28 protein has been found in all cell types of the adult (Galy *et al.* 2006). Yet, *mel-28* is a strict embryonic lethal gene; homozygous *mel-28* animals survive to adulthood as long as they receive maternally provided *mel-28* product *in utero*. Such animals grow up to produce a brood size comparable with the wild type (this study) but none of their embryos are viable. Maternal-effect lethal genes are thought to have critical function during early embryogenesis that is no longer required in the adult. Because *mel-28* functions in processes that are important in all cells, we hypothesized that there are other genes that act in concert with *mel-28* and that can compensate for its loss in cells of the *mel-28* mutant adult.

One goal of our screen was to unmask a role for *mel-28* in postembryonic development. We also sought to identify processes that might work in partnership with the nuclear envelope or with chromosome segregation. To accomplish these goals, we performed an RNAi screen seeking genes that cause phenotypes in *mel-28* but not wild-type (N2) animals.

## Materials and Methods

### Worm strains

#### RNAi screen:

The RNAi screen was performed in 96-well plates as described in Fernandez *et al.* (2010) and Cipriani and Piano (2011). We administered RNAi by feeding, using publically available bacterial RNAi clone libraries (Kamath *et al.* 2003; Rual *et al.* 2004). In summary, 0.5 ml of RNAi cultures were grown overnight in 96-well plates, induced for 3 hr using 1 mM IPTG, then pelleted and resuspended in 0.5 mL S medium supplemented with 100 μg/mL ampicillin and 1 mM IPTG. Twenty microliters of each resuspension was dispensed into the equivalent position of each of three new 96-well plates. To collect large quantities of homozygous *mel-28* L1 larvae, we used a method called laFACS, in which a FACS machine is used to sort and collect large quantities of live worms of a specific genotype (Fernandez *et al.* 2010, 2012). Once collected, *mel-28* homozygotes from strain PF405 were diluted in S medium + 100 μg/mL ampicillin + 1 mM IPTG to a concentration of 5−10 L1 larvae per 30 μL, then 30 μL of worm resuspension was added to each well. For each of the three RNAi plate replicas, one plate received N2 L1s and two plates received *mel-28* L1s. Thus, for each experimental run, a given RNAi clone was tested in one pool of N2 animals and two pools of *mel-28* animals. Two 96-well plates containing only control RNAi bacteria (L4440) were run with each RNAi experiment. One of these plates was loaded with N2 L1s and the other with *mel-28* L1s. RNAi plates were placed in a humid chamber at 20°. After 6−8 d, wells were photographed using a Leica Z16 dissecting scope fitted with a DFC340 FX camera and Surveyor software was used to automate the camera and the stage.

We tested all viable RNAi clones from the Ahringer lab RNAi library (Kamath *et al.* 2003) and nonoverlapping clones from the Vidal lab RNAi library (Rual *et al.* 2004). A total of 18,364 clones from both libraries were tested. RNAi images were qualitatively scored. In a pilot screen (Fernandez *et al.* 2010), we found that sterility was a phenotype that could be easily and reproducibly scored in high throughput; thus, we used synthetic sterility as a readout for a potential genetic interaction. To reduce the number of false negatives arising from our first filtering step, we elected to retain many clones for further testing. Clones that (1) showed any fertility difference between *mel-28* and N2 animals or (2) produced wells that could not be evaluated for technical reasons (such as not enough worms were added) were rearrayed and tested in five additional RNAi experiments. There were 884 clones that met these criteria and were retested. These were first qualitatively scored for differences between *mel-28* and N2 trials. On the basis of these qualitative scorings, the RNAi images from 181 clones were quantitatively reassessed. N2 and *mel-28* animals treated with control RNAi bacteria produce a range of 20 ± 7 observable progeny per adult in our assays. Where the RNAi treatment caused more than 13 progeny per adult in N2 trials and 0−3 eggs per adult in *mel-28* trials, we declared the genetic interactor “synthetic sterile” with *mel-28*. Where the RNAi clone caused 2−14 larvae per N2 adult and 0 ± 3 eggs per *mel-28* adult we called the genetic interaction “enhancement.” Most of the genes we identified caused complete sterility in *mel-28* animals, and for all genetic interactors the N2 brood size was at least three times larger in N2 compared with *mel-28* animals. From the quantitative analysis, we identified 81 clones that reproducibly caused severe brood size reduction in *mel-28* animals but not N2 animals. All 81 clones were sequenced. After sequencing, four of the bacterial stocks could not be unambiguously assigned to a single gene and were thus excluded from the study. The remaining 77 clones corresponded to 73 unique genes.

As a final RNAi filtering step, we retested RNAi clones corresponding to the 73 unique genes on solid medium, using *mel-28* mutants manually selected from the AGF001 strain (that no longer carries the linked *unc-32* allele). In these tests, we found eight clones that no longer cause a synthetic phenotype with *mel-28*. These eight genes were removed from our list of *mel-28* genetic interactors, leaving 65 genes. Representative photos of RNAi experiments in N2 and *mel-28* with each of the 65 genes are shown in Supporting Information, Figure S1. We present brood size data for N2 and *mel-28* trials for each genetic interactor we identified in Table S1.

One clone representing a *mel-28* interactor (the *dhc-1* gene) was tested in only one RNAi experiment. This clone was not selected for retests after the initial screening because it caused near sterility in the N2 trial. After recovering other genes related to dynein function from the screen, we reanalyzed captured images from the initial *dhc-1* RNAi experiment and found that indeed it showed a weak genetic interaction with *mel-28* via our assays. This genetic interaction was later confirmed via double-mutant analysis (see the section *Double-mutant analysis*).

### Double-mutant analysis

We generated study strains (see Table 1) that carried a *qC1*-balanced *mel-28* allele and were also homozygous for the putative genetic interactor. *mel-28* heterozygotes were identified by their roller phenotype (conferred by the *rol-6^D^* allele on *qC1*) and homozygous *mel-28* animals by their nonroller phenotype. We used a version of the *qC1* balancer that carries a recessive lethal allele; thus, all live animals derived from these strains are either *mel-28* heterozygotes (and roller) or *mel-28* homozygous (and nonroller). These were compared with each other and to rollers and nonrollers from the AGF001 strain. Twelve L4 animals of each genotype were individualized and moved to new plates daily. The total number of eggs laid for each animal was tallied. Animals were also monitored daily for viability. Lifespan and brood size averages were compared using a two-tailed Student’s *t*-test. Strains carrying *npp-11*, *npp-12*, and *npp-14* were maintained and analyzed at 20°, the *dnc-1* and *dhc-1* strains (which carry temperature-sensitive alleles) were maintained at 15° and shifted to 25° at the L4 stage for analysis and the *npp-5* strain (which is very sick at higher temperatures) was maintained and analyzed at 15°.

**Table 1:** Strains used in this study

Strain Name	Genotype	Purpose
PF405	*mel-28(t1684) unc-32(e189)*/qC1 *dpy-19(e1259) glp-1(q339)* [qIs26] III	Initial RNAi screen
AGF001	*mel-28(t1684)*/qC1 *dpy-19(e1259) glp-1(q339)* [qIs26] III	RNAi screen−positive clone retests, controls in double-mutant analyses
AGF034	*mel-28(t1684)/qC1 dpy-19(e1259) glp-1(q339)* [qIs26] III; *npp-12(ok2424)* I	*mel-28;npp-12* double-mutant analysis
AGF035	*mel-28(t1684)/qC1 dpy-19(e1259) glp-1(q339)* [qIs26] III;*dhc-1(or283ts)* I	*mel-28;dhc-1* double-mutant analysis
AGF037	*mel-28(t1684)/qC1 dpy-19(e1259) glp-1(q339) [qIs26] III;npp-11(ok1599)* I	*mel-28;npp-11* double-mutant analysis
AGF038	*mel-28(t1684)/qC1 dpy-19(e1259) glp-1(q339)* [qIs26] III;*npp-5(ok1966)* II	*mel-28;npp-5* double-mutant analysis
AGF044	*mel-28(t1684)/qC1 dpy-19(e1259) glp-1(q339*) [qIs26] III; *npp-14(ok1389)* I	*mel-28;npp-14* double-mutant analysis
AGF046	*mel-28(t1684)/qC1 dpy-19(e1259) glp-1(q339)* [qIs26] III; *dnc-1(or404)* IV	*mel-28;dnc-1* double-mutant analysis

RNAi, RNA interference.

## Results

### General trends

*mel-28* is a conserved and essential gene that is expressed throughout the *C. elegans* cell lineage. Yet, as it is a strict maternal-effect lethal gene, its requirement for viability is apparent only in the embryo. Thus the essential roles MEL-28 has in the embryo appear to be buffered or compensated for in other cells, making *mel-28* a candidate to uncover cell-specific buffering using a genome-scale strategy.

**Figure 1:**
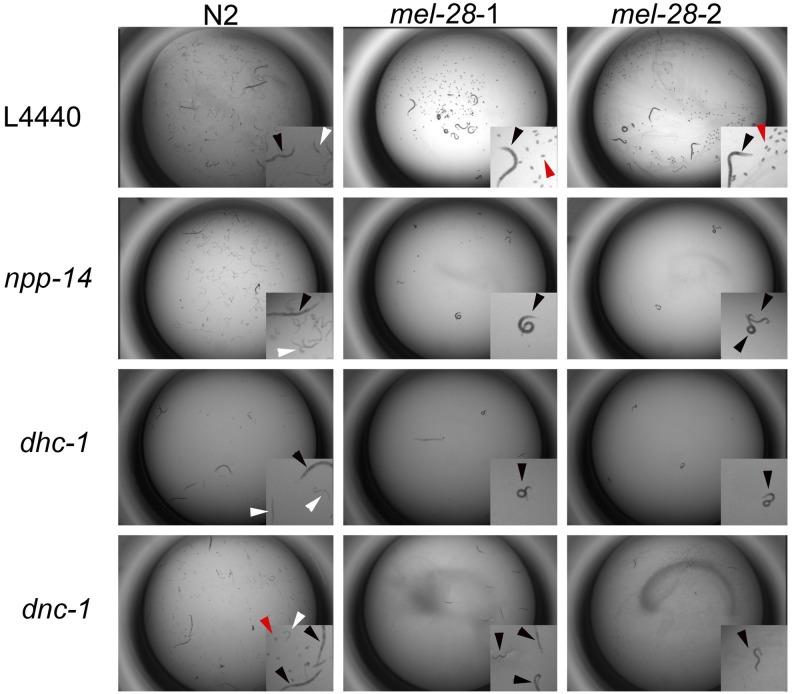
Representative genetic interaction phenotypes. The N2 trial and both *mel-28* trials are shown for each RNAi experiment. L4440 is the empty vector control. On the L4440 control, N2 animals produce many eggs that hatch and *mel-28* animals produce many eggs that fail to hatch (see insets). *npp-14* and *dnc-1* RNAi treatments do not affect the brood size of *mel-28* animals, but they cause sterility (no eggs) in *mel-28* animals. These are synthetic sterile interactions. *dhc-1* RNAi treatment causes a reduced brood size in N2 animals and sterility in *mel-28* animals. This phenotype represents enhancement. The black arrowheads indicate adult animals, the white arrowheads point to larvae, and the red arrowheads indicate embryos.

To identify *mel-28* genetic interactors, we used RNAi to disrupt gene function in wild-type (N2) and *mel-28* mutant animals. We sought genes that caused severe brood size defects in *mel-28* but not N2 worms. In addition, we looked for genes whose disruption rescues the embryonic lethality caused by the *mel-28* defect. After filtering our results for reproducibility across several trials (see *Materials and Methods*) we found 65 genes that produce severe brood size reduction in *mel-28* mutants compared with the N2. (see Figure 1 and Figure S1 for representative images from the experiments). We name these genes “*mel-28* genetic interactors,” and these are listed in Table 2 and Table S1.

**Table 2:** *mel-28* genetic interactors

Gene	Predicted Protein (Wormbase WS238)	Genetic Interaction
Cell attachments		
* pat-2*	α-integrin subunit	Synthetic sterility
* pat-6*	α-parvin (actopaxin) homolog	Enhancement
* klf-3*	Predicted transcription factor that affects cell-matrix attachments	Enhancement
Chromatin		
* his-67*	Histone H4	Enhancement
* his-68*	Histone 2A	Enhancement
* pyp-1*	Inorganic pyrophosphatase implicated in nucleosome remodeling	Enhancement
* ruvb-2*	Potentially involved in transcription regulation via chromatin remodeling	Enhancement
Dynein/dynactin		
* dhc-1*	Dynein heavy chain	Enhancement
* dyci-1*	Dynein intermediate chain	Enhancement
* dli-1*	Dynein light intermediate chain	Synthetic sterility
* dnc-1*	p150/Glued component of dynactin	Synthetic sterility
* cap-1*	F-actin capping protein, α subunit, dynactin component	Enhancement
* cap-2*	F-actin capping protein, β subunit, dynactin component	Enhancement
* arp-1*	Actin related protein, dynactin component	Synthetic sterility
Nuclear envelope		
* npp-2*	Nup75 nuclear pore component	Enhancement
* npp-4*	Nup98-96 nuclear pore component	Synthetic sterility
* npp-5*	Nup107 nuclear pore component	Synthetic sterility
* npp-12*	Gp210 nuclear pore component	Synthetic sterility
* npp-14*	Nup214 nuclear pore component	Synthetic sterility
* npp-15*	Nup133 nuclear pore component	Synthetic sterility
* npp-17*	Rae1 nuclear pore component	Enhancement
* npp-20*	Sec13R nuclear pore component	Enhancement
* npp-22*	Ndc1 nuclear pore component	Synthetic sterility
* ima-3*	Importin α nuclear transport factor	Enhancement
* vrk-1*	Vaccinia-related kinase required for nuclear envelope formation	Enhancement
* lpin-1*	Putative phosphatidic acid phosphatase required for nuclear envelope breakdown	Enhancement
Protein chaperone		
* cct-2*	Putative β subunit of the eukaryotic cytosolic chaperonin	Synthetic sterility
* stc-1*	hsp70 homolog	Enhancement
* sca-1*	Sarco-endoplasmic reticulum Ca^2+^-dependent ATPase	Enhancement
Protein sorting/ vesicle trafficking		
* phi-56*	Endoplasmic reticulum signal peptidase	Enhancement
* K12H4.4*	Subunit of signal peptidase complex	Enhancement
* ggtb-1*	GeranylGeranyl transferase β subunit	Enhancement
* ykt-6*	Synaptobrevin vesicle membrane protein (v-SNARE)	Enhancement
* syx-4*	Syntaxin-related (t-SNARE)	Synthetic sterility
* mua-6*	Intermediate filament involved in ER-to-Golgi SNARE complex	Synthetic sterility
* syd-9*	Zinc-finger protein that regulates synaptic vesicle endoctyosis	Synthetic sterility
* arf-3*	ADP-ribosylation factor related	Enhancement
* hgrs-1*	Homologous to *S. Cerevisiae* Vps27p	Synthetic sterility
* vps-32.2*	Homologous to *S. Cerevisiae* SNF7p	Enhancement
Translation		
*Y61A9LA.10*	GTPase involved in assembly of 40S ribosomal subunits	Enhancement
*eif-1*	Eukaryotic translation initiation factor	Enhancement
Proteasome		
*Y39C12A.1*	Uncharacterized protein with homology to Nas6p, a proteasome- interacting protein from *S. Cerevisiae*	Synthetic sterility
*F52C6.2*	Uncharacterized protein with homology to ubiquitin	Synthetic sterility
*F52C6.3*	Uncharacterized protein with homology to ubiquitin	Synthetic sterility
RNA regulation		
* exos-3*	Multiexonuclease complex component involved in RNA processing	Enhancement
* alg-1*	Homologous to argonaute	Enhancement
Other functions		
* cyd-1*	Cyclin important for G1/S transition	Synthetic sterility
* ego-2*	Bro1 domain protein	Synthetic sterility
* dre-1*	F-box protein with roles in developmental timing	Enhancement
* nipi-3*	Kinase with function in innate immune response	Enhancement
* hmgs-1*	Mevalomate biosynthesis	Synthetic sterility
* nhr-25*	Nuclear hormone receptor homologous to *Drosophila* Ftz-F1	Synthetic sterility
* nhr-67*	Nuclear hormone receptor homologous to *Drosophila* Tailless	Synthetic sterility
* apl-1*	Amyloid β A4 precursor protein	Synthetic sterility
*F19B6.1*	Uridine-cytidine kinase-like, acts in the ribonucleotide salvage pathway	Synthetic sterility
* C55A6.9*	Homologous to Pad1p complex component in yeast, which regulates RNA Polymerase I and II activity	Enhancement
* egl-13*	Sox domain transcription factor	Synthetic sterility
* sox-2*	Sox domain transcription factor	Synthetic sterility
gei-13	BED-domain protein, predicted to bind to DNA	Synthetic sterility
* Y53F4B.13*	uncharacterized	Enhancement
*lin-3*	EGF family peptide growth factor with roles in germ-line development	Synthetic sterility
*arx-2*	Component of Arp2/3 complex, an actin nucleation center	Synthetic sterility
*eat-6*	Alpha subunit of a sodium/potassium atpase	Enhancement
*B0250.7*	Noncoding transcript	Enhancement
*sos-1*	Homolog of *son of sevenless*, which encodes a guanine nucleotide exchange factor	Enhancement

EGF, epidermal growth factor.

We did not identify suppressors of the *mel-28* phenotype (*i.e.*, genes that allow embryos from homozygous *mel-28* animals to hatch), which may be because MEL-28 is critical structural component in the postmitotic rebuilding of the nuclear pore, as is suggested by studies of ELYS in vertebrate systems (Franz *et al.* 2007; Rasala *et al.* 2008). In addition the *t1684* allele we used is an early nonsense mutation and thus a likely null. As we screened an estimated 85% of the genome, it is likely that there are no genes whose disruption by RNAi compensates for the complete loss of *mel-28* function in the embryo.

Each of the genes we identified produced a reproducible phenotypic difference between N2 and *mel-28* animals. In addition, we retested some genes via double-mutant analysis (see *Double-mutant confirmation of RNAi results*), and all these experiments confirmed the results we observed via RNAi. Therefore, there is no evidence of false positive data in our results. However, given that RNAi effectiveness can vary and that we did not screen about ~15% of the genome, there may be additional *mel-28* genetic interactors that we did not identify here.

### *mel-28* genetic interactors

Among the 65 genes we found in our screen, there were several categories of gene function that were represented multiple times. We used FuncAssociate (http://llama.mshri.on.ca/funcassociate_client/html/) to query this list for GO term enrichment, and we found 55 GO terms significantly overenriched within this set. A complete list of enriched GO terms is in Table S2. All genes identified are listed in Table 2.

#### Nuclear envelope:

**Figure 2:**
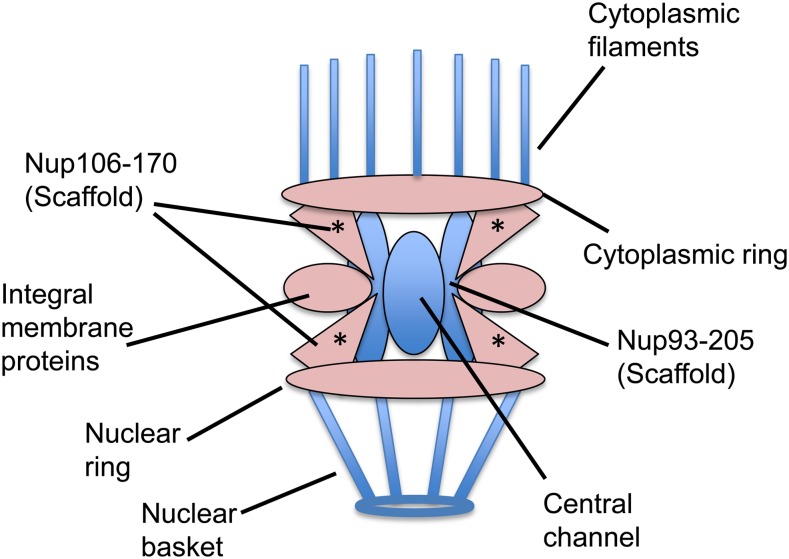
The nuclear pore is composed of multiple subcomplexes (illustration adapted from D’Angelo and Hetzer 2008). Subcomplexes colored pink have components that cause synthetic sterility when depleted in *mel-28* animals. Subcomplexes colored blue have no components that show a genetic interaction with *mel-28*. The asterisk marks the Nup107-160 subcomplex, with which ELYS/ MEL-28 directly interacts in HeLa cells and *Xenopus* extracts (Franz *et al.* 2007; Rasala *et al.* 2008).

Of the 23 known nucleoporin genes in *C. elegans*, we identified nine that are *mel-28* genetic interactors (Table 3). Four of the eight genes that encode components of the Nup107-160 scaffolding complex (with which ELYS, the vertebrate homolog of MEL-28 directly interacts; Franz *et al.* 2007; Rasala *et al.* 2008) were found. We also found genes encoding integral membrane proteins of the nuclear pore and components of the cytoplasmic and nuclear rings (Figure 2).

**Table 3:** Genetic interaction tests with *mel-28* and nuclear pore complex components

Gene	Protein*^a^*	Location (Vertebrate Systems)*^b^*	*mel-28* Interactor?	Comments
*npp-1*	Nup54	Central channel	No	Sterile in N2
*npp-2*	Nup75	Nup107-160 scaffold	Yes	
*npp-3*	Nup205	Nup93-205 scaffold	No	Sterile in N2
*npp-4*	Nup96-98	Nuclear and cytoplasmic rings	Yes	
*npp-5*	Nup107	Nup107-160 scaffold	Yes	
*npp-6*	Nup160	Nup107-160 scaffold	No	Fertile in all trials
*npp-7*	Nup153	Nuclear basket	No	Fertile in all trials
*npp-8*	Nup155	Nup93-205 scaffold	Not tested	
*npp-9*	Nup358	Cytoplasmic filament	No	Sterile in N2
*npp-10*	Nup98	Nup107-160 scaffold	No	Sterile in N2
*npp-11*	Nup62	Central channel	No	Fertile in all trials
*npp-12*	Gp210	Integral membrane	Yes	
*npp-13*	Nup93	Integral membrane	Not tested	
*npp-14*	Nup214	Cytoplasmic ring	Yes	
*npp-15*	Nup133	Nup107-160 scaffold	Yes	
*npp-16*	Nup50	Nuclear ring	No	Fertile in all trials
*npp-17*	Rae1	Nuclear and cytoplasmic rings	Yes	
*npp-18*	Seh1	Nup107-160 scaffold	No	Fertile in all trials
*npp-19*	Nup35	Nup93-205 scaffold	No	Fertile in all trials
*npp-20*	Sec13R	Nup107-160 scaffold	Yes	
*npp-21*	TPR	Nuclear basket	No	Fertile in all trials
*npp-22*	Ndc1	Integral membrane	Yes	
*npp-23*	Nup43	Nup107-160 scaffold	No	Fertile in all trials

a From Galy *et al.* 2003 and WS 238.

b From D’Angelo and Hetzer 2008 and Neumann *et al.* 2010.

Another nuclear envelope−associated gene we found is *vrk-1*, which encodes vaccinia-related kinase and regulates nuclear envelope formation via phosphorylation of BAF-1 (Gorjanacz *et al.* 2007). In addition, *lpin-1*, which encodes a crucial regulator of nuclear envelope disassembly, also was found in this screen (Golden *et al.* 2009; Gorjanacz and Mattaj 2009). We also identified *ima-3*, which encodes a regulator of nucleocytoplasmic exchange. This gene is required for proper assembly of the nuclear pore and progression through meiotic prophase I during oocyte development (Geles and Adam 2001).

#### Dynein/dynactin:

**Figure 3:**
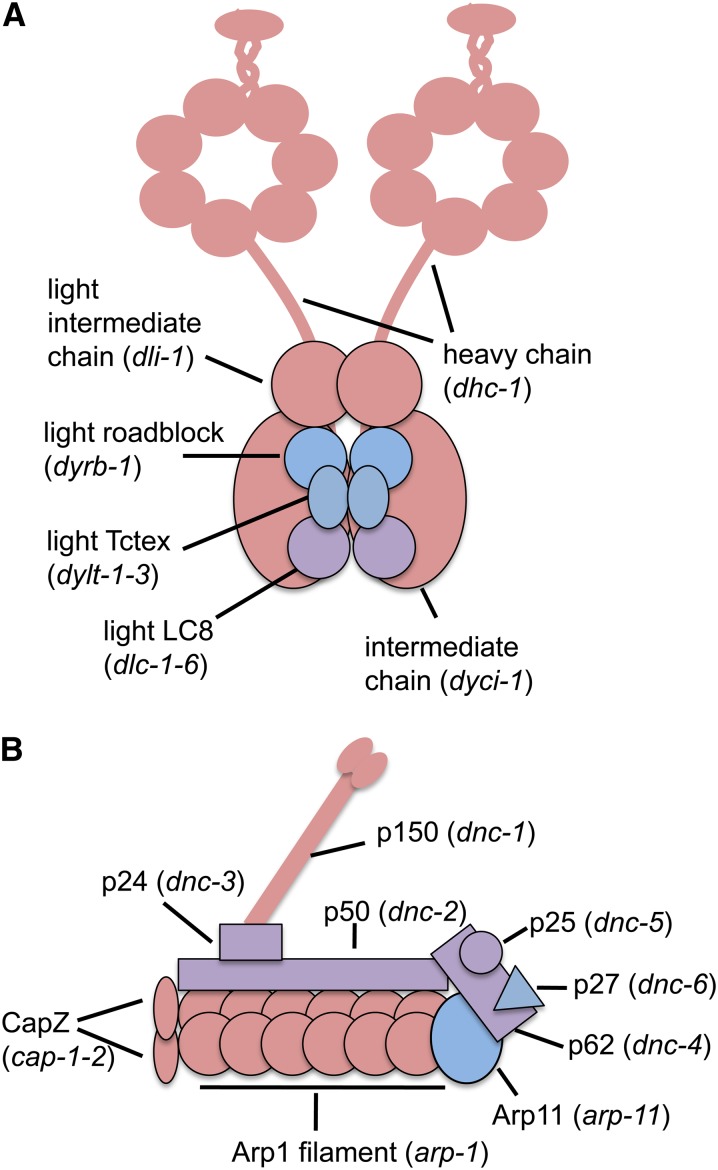
Dynein (A) and dynactin (B) are composed of multiple polypeptides (illustrations adapted from Pfister *et al.* 2006 and Terasawa *et al.* 2010). Components colored pink cause synthetic sterility with *mel-28*, those colored blue did not, and those colored lavender were not tested. Each component is labeled, and the *C. elegans* gene that encodes that polypeptide is shown in parentheses.

Dynein is a multisubunit minus end−directed microtubule motor implicated in several processes, including nuclear envelope breakdown, chromosome segregation, vesicle trafficking, and nuclear positioning (Dujardin and Vallee 2002). Genes encoding the three largest subunits of dynein were recovered from our screen (Figure 3). We also recovered genes that encode four components of dynactin, a complex required for dynein to target its cargo (Figure 3) (Schroer 2004).

### Cell attachments

*pat-6* is an actoplastin homolog implicated in focal adhesions and is required for integrin-mediated body wall muscle attachments (Lin *et al.* 2003). *pat-2* encodes the α subunit of transmembrane structures that connect actin and myosin filaments to the cell membrane (Rogalski *et al.* 2000). The PAT-2/PAT-3 heterodimer is found in most contractile tissues of the worm. *klf-3* encodes a transcription factor that regulates muscle-specific genes (Himeda *et al.* 2010). In *C. elegans*, *klf-3* mutants display muscle detachment phenotypes (Plenefisch *et al.* 2000).

#### Chromatin:

*his-67* and *his-68* encode histone H4 and histone 2A, components of the nucleosome (WormBase WS 238). *pyp-1* encodes an inorganic pyrophospahatase homologous to NURF-38 in Drosophila, a protein implicated in chromatin remodeling (Gdula *et al.* 1998). *ruvb-2* encodes an AAA+ ATPase that influences multiple processes, including chromatin remodeling (Jha and Dutta 2009). Perhaps in the germline *mel-28* redundantly contributes to the chromatin morphology changes necessary for proper progression through prophase I. When other factors that regulate chromatin state are compromised, *mel-28* function becomes required for the formation of gametes.

#### Protein chaperones:

*cct-2* encodes a protein chaperone necessary for the proper biogenesis of tubulin and likely other proteins too (Lundin *et al.* 2008). *stc-1* mutants accumulate misfolded proteins, and *stc-1* encodes an endoplasmic reticulum (ER)-localized Hsp70 homolog (Wang *et al.* 2009). *sca-1* encodes a SERCA, a Sarco(endo)plasmic reticulum calcium ATPase that regulates cytsosolic calcium by pumping it into the ER (Zwaal *et al.* 2001). RNAi of *sca-1* in *C. elegans* causes contractile dysfunctioning, likely attributable to defects in organization of myofilaments (Zwaal *et al.* 2001; Meissner *et al.* 2009). Disruption of either *sca-1* or *stc-1* causes compensatory up-regulation of *hsp-4*, an ER chaperone (Kapulkin *et al.* 2005), suggesting that both genes encode ER resident chaperones. It may be that misfolded proteins accumulate in *mel-28* mutants, sensitizing these animals to defects in protein chaperones. Many of these chaperones are necessary for the proper assembly of cytoskeletal elements. Thus, another possibility is that *mel-28* mutants require the efficient organization of the cytoskeleton within the developing gonad in order to produce functioning gametes.

#### Protein sorting/vesicle trafficking:

*phi-56* is homologous to the 12-kDa component of the ER signal peptidase, which is required for cleaving the N-terminal tag of ER-bound polypeptides and also plays a role in the ER-mediated degradation of abnormal proteins (Mullins *et al.* 1995). *K12H4.4* is homologous to the SPC22/23 component of the signal peptidase complex (WormBase WS238). *ggtb-1* encodes the beta subunit of the gernaylgeranyl transferase, which adds the gernaylgeranyl moiety to target proteins, allowing them to associate with membranes (Mukhopadhyay *et al.* 2007). Mutations affecting this subunit in Arabidopsis have defects in exocytosis and endocytosis (Hala *et al.* 2010). *vps-32.2* encodes a homolog of endosomal sorting complex ESCRT-III from *S. cerevisiae*, which is involved in the sorting of membrane-bound proteins into endosomes (Babst *et al.* 2002). We identified several genes relating to SNARE complex proteins, which are soluble *N*-ethylmaleimide−sensitive factor attachment protein receptors needed for vesicle docking (Carr and Rizo 2010). *mua-6* encodes a cytoplasmic intermediate filament important for the muscle to hypodermis attachment (Hapiak *et al.* 2003). The yeast homolog of *mua-6*, USO1, is required for vesicle transport between the ER and the Golgi and in fact is required specifically for the vSNARE and tSNARE docking complex assembly needed for vesicle fusion (Sapperstein *et al.* 1996). *ykt-6* encodes v-SNARE, which is a vesicle-localized protein that mediates fusion of vesicles to a target membrane (Nagahama *et al.* 1996; Subramaniam *et al.* 1996). *syx-4* encodes tSNARE, which is the receptor in the target membrane that vSNARE contacts in order for membrane fusion to occur (Jantsch-Plunger and Glotzer 1999). *syd-9* mutants have defects in synaptic transmission and specifically in endocytosis of synaptic vesicles (Wang *et al.* 2006). *arf-3* encodes an ADP ribosylation factor, a protein that coats vesicles and is required for vesicular transport. (Serafini *et al.* 1991). *hgrs-1* encodes an ortholog of *S. cerevisiae* Vps27p, and is required for endocytic trafficking in *C. elegans* (Roudier *et al.* 2005).

#### Translation:

*Y61A9LA.10* encodes a GTPase homologous to a yeast protein BMS1, which is required for synthesis and processing of ribosomal RNAs (Wegierski *et al.* 2001; Karbstein *et al.* 2005). *eif-1* encodes a translation initiation factor necessary for regulation of start site selection during the initiation of translation (Mitchell and Lorsch 2008). It is not clear why loss of *mel-28* function enhances the reduced brood size defects caused by genes involved in translation initiation. However, there are previously established links between gonadogenesis and ribosome biogenesis in *C. elegans* (Voutev *et al.* 2006; Kudron and Reinke 2008).

#### Proteasome:

*Y39C12A.1* encodes a homolog of Nas1p, a subunit of the 26S proteasome (Hori *et al.* 1998). *F52C6.2* and *F52C6.3* both encode homologs of ubiqutin, which tag proteins for degradation via the 26S proteasome (Pickart 1997).

#### RNA regulation:

*exos-3* encodes a component of the exosome that is required for RNA surveillance. *exos-3* is induced by ER stress and disruption *of exos-3* causes ER stress (Sakaki *et al.* 2012). *alg-1* is an *argonaute* homolog that is required for the processing of small RNAs that affect the heterochronic pathway in *C. elegans* (Grishok *et al.* 2001).

#### Other:

We identified 19 other *mel-28* genetic interactors that do not have an obvious connection to the above classes, nor an obvious connection to each other (Table 2).

### Double-mutant confirmation of RNAi results

To better quantify the defects caused by the simultaneous disruption of *mel-28* and some of the *mel-28* genetic interactors, we generated double mutants. To do this, we made a *qC1*-balanced *mel-28* strain homozygous for each *mel-28* genetic interactor (see *Materials and Methods*), which allowed us to compare *mel-28* heterozygotes with *mel-28* homozygotes from the same strain.

**Figure 4:**
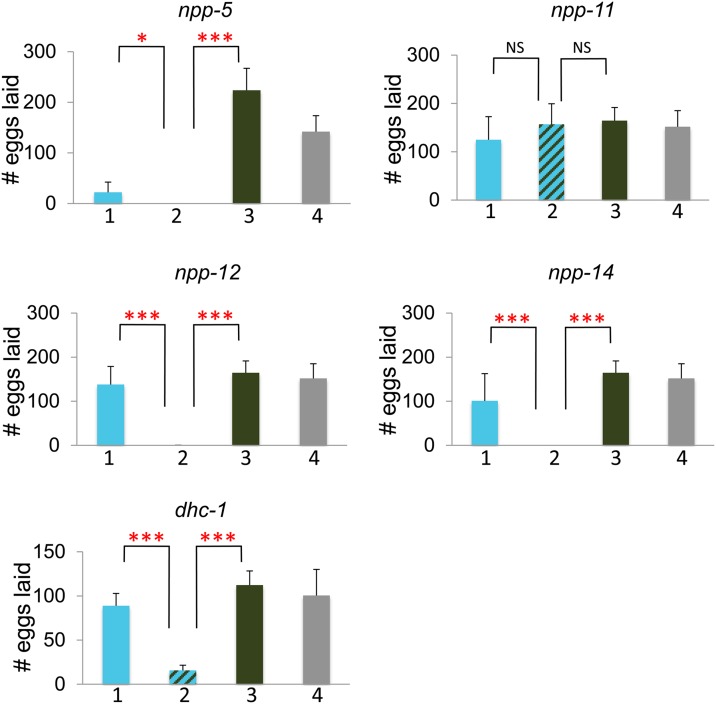
*mel-28* genetic interactions produce brood size defects. The Y-axis indicates number of eggs laid. Bar 1 (in blue) represents animals homozygous for the putative genetic interactor and heterozygous for *mel-28*, bar 2 (striped) represents animals homozygous for both *mel-28* and the putative genetic interactor, bar 3 (green) represents animals homozygous for *mel-28* but otherwise wild type, and bar 4 (gray) represents animals heterozygous for *mel-28* but otherwise wild type. See *Materials and Methods* for experimental details. The asterisks indicate the significance of the difference between the double mutant and each single mutant (*P < 0.05; **P < 0.01; ***P < 0.001; NS, not significant).

**Figure 5:**
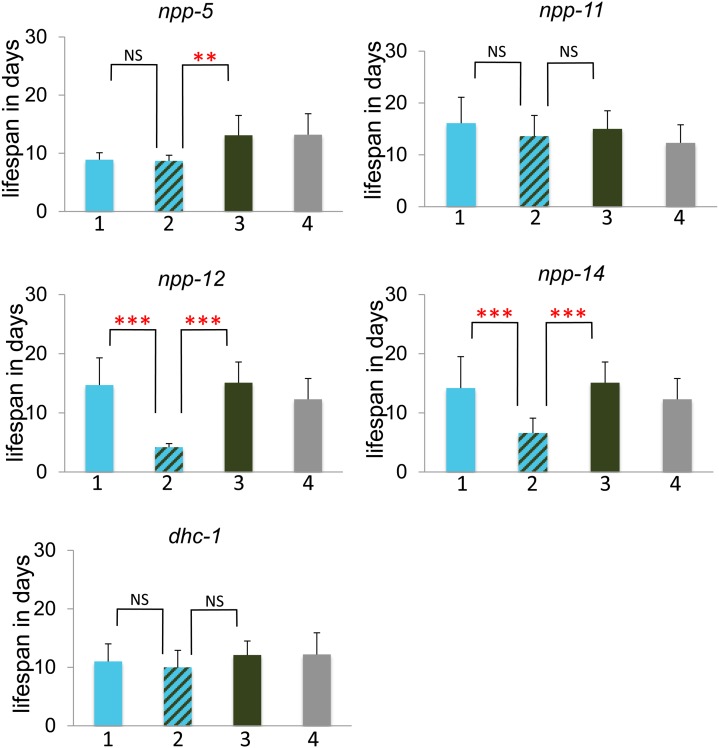
Some *mel-28* genetic interactions produce lifespan defects. The Y-axis indicates lifespan in days. Bar 1 (in blue) represents animals homozygous for the putative genetic interactor and heterozygous for *mel-28*, bar 2 (striped) represents animals homozygous for both *mel-28* and the putative genetic interactor, bar 3 (green) represents animals homozygous for *mel-28* but otherwise wild type, and bar 4 (gray) represents animals heterozygous for *mel-28* but otherwise wild type. See *Materials and Methods* for experimental details. The asterisks indicate the significance of the difference between the double mutant and each single mutant (*P < 0.05; **P < 0.01; ***P < 0.001; NS, not significant).

To further investigate the genetic interactions between *mel-28* and other nuclear pore components, we studied *mel-28*; *npp-5*, *mel-28*; *npp-11*, *mel-28*; *npp-12*, and *mel-28*; *npp-14* double mutants. *npp-5*, *npp-12*, and *npp-14* mutations produced 100% sterility in *mel-28* mutants (Figure 4). *npp-14;mel-28*, and *npp-12;mel-28* double mutants also showed a significantly reduced lifespan compared with each single mutant (Figure 5). *npp-5* homozygotes individually have reduced brood size and viability, but the brood size defect is significantly worse in the *npp-5;mel-28* double mutants (which are sterile) (Figure 4). Each of these genes represents a different subcomplex of the nuclear pore (see Table 3). The *npp-11;mel-28* double mutants showed no additional defects compared to each single mutant (Figure 4 and 5), which is consistent with our RNAi study (which did not identify *npp-11* as a *mel-28* genetic interactor). Thus, these double-mutant analyses confirmed the results of the RNAi screen; only those components identified as a genetic interactor from the RNAi screen exhibited synthetic sterility with *mel-28* via double mutant analyses.

We also investigated double mutant phenotypes with dynein and dynactin components. The *dhc-1* RNAi experiment showed a weak genetic interaction with *mel-28* (Figure 1). This interaction was confirmed via double mutant analysis (Figure 4). We used a temperature sensitive (*or283ts*) allele of *dhc-1* and observed a significantly smaller brood size in *dhc-1;mel-28* double mutants than in either single mutant when we grew the animals at 25°. Lifespan of the double mutants was unaffected (Figure 5). Confirmation of this weak interaction via double mutant analysis shows that the relatively subtle phenotype we observed in the screen is a genuine consequence of the simultaneous disruption of *mel-28* and *dhc-1*.

**Figure 6:**
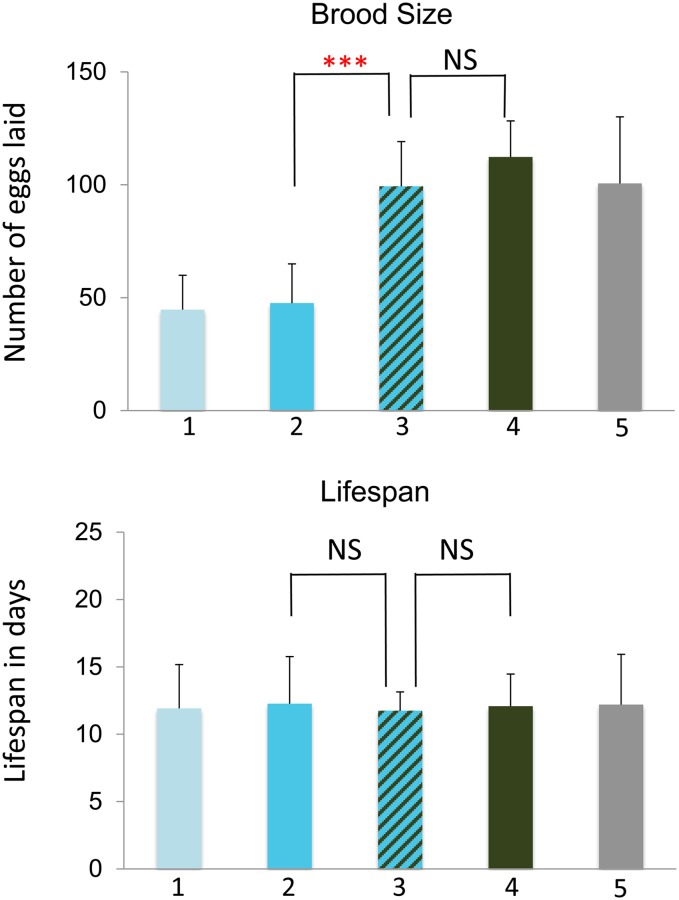
*mel-28* mutations rescue the small brood size defect caused by *dnc-1(or404*) at 25° but do not affect lifespan. Bar 1 (pale blue) represents animals homozygous for *dnc-1(or404)* and otherwise wild type, bar 2 (sky blue) represents animals homozygous for *dnc-1(or404*, and heterozygous for *mel-28*, bar 3 (striped) represents animals homozygous for both *mel-28* and *dnc-1(or404)*, bar 4 (green) represents animals homozygous for *mel-28* but otherwise wild type, and bar 5 (gray) represents animals heterozygous for *mel-28* but otherwise wild type. See *Materials and Methods* for experimental details. The asterisks indicate the significance of the difference between the double mutant and each single mutant (*P < 0.05; **P < 0.01; ***P < 0.001; NS, not significant).

*dnc-1* encodes the large subunit of the dynactin complex, which is required to couple the dynein motor to its cargo (Schroer 2004; Kardon and Vale 2009). We used the temperature-sensitive *or404* allele of *dnc-1* in the double mutant analyses with *mel-28*, and we moved L4 animals to 25° for analysis. *dnc-1(or404)* homozygotes produce a small brood size in these conditions. In the same conditions, the *dnc-1;mel-28* double mutants produced a normal brood size (Figure 6). This result confirms that there is a genetic interaction between *dnc-1* and *mel-28*. However, the *dnc-1* RNAi treatment causes sterility in *mel-28* animals but not N2 (Figure 1). One explanation for this difference between the RNAi result and the double mutant analysis is that the *dnc-1(or404)* allele is not a null. In fact, this mutation causes a threonine to cysteine amino acid substitution at position 1237 of the a isoform (WormBase WS 238). We used RNAi to deplete *dnc-1* from *mel-28;dnc-1(or404)* double mutants, and we found that this treatment caused a much stronger phenotype: the animals died before producing eggs (our unpublished results). This indicates that the brood-size rescue observed in *dnc-1(or404);mel-28* double mutants is dependent upon active DNC-1 protein being available; reducing the pool of DNC-1 using RNAi prevents rescue.

## Discussion

The goal of this work was to identify genes that contribute coordinately to development with *mel-28*. In addition we sought to identify novel postembryonic roles for *mel-28* and to use this as a case study to uncover tissue-specific genetic networks. We accomplished these goals by doing an RNAi-based genetic interaction screen seeking genes that cause a phenotype in *mel-28* animals but not wild-type worms.

Some of the *mel-28* genetic interactors we identified here might have general roles in protecting the organism from perturbation and thus do not relate specifically to *mel-28* function. For example, we found three genes relating to protein chaperone activity, and protein chaperones are promiscuous buffers of phenotypic variation (Rutherford 2003).

The relatively weak knockdown achieved with RNAi by feeding allowed us to interrogate genes which, by themselves, induce a sterile or reduced viability phenotype as a stronger knockdown. This is similar to a strategy of using suboptimal morpholino injections to create a sensitized background for the discovery of synergistic interactions between genes (DiBella *et al.* 2009). Some of the genes (*e.g.*, *lpin-1* and *dhc-1*) that we identified as *mel-28* genetic interactors were reported previously to cause sterility when individually RNAi-depleted by soaking (Green *et al.* 2008), which tends to cause a stronger knockdown (Sugimoto 2004; Fernandez *et al.* 2005). Thus, the weaker RNAi depletion strategy we used allowed us to uncover genetic interactions that might otherwise have been difficult or impossible to observe.

Ultimately, we identified 65 *mel-28* genetic interactors. Most of these genes fall into several major classes of gene function (Table 2). Here, we discuss the three classes that include the largest number of genes.

### Protein sorting/vesicle trafficking

The maturation of germ cells within the *C. elegans* germline requires a constant reorganization of cell membranes. The *C. elegans* gonad consists of two U-shaped syncytial tubes called gonad arms that have a distal to proximal polarity. The distal tip of each gonad arm is a stem cell niche in which the nuclei divide mitotically and are undifferentiated (Hirsh *et al.* 1976; Kimble and White 1981) As nuclei progress from distal to proximal, they enter meiosis and progress through the stages of prophase I. Within the gonad, the nuclei are partially enclosed by cell membranes but remain open to a central lumen (Hirsh *et al.* 1976). During the last larval stage of development, nuclei that have progressed to the proximal end of each gonad arm cellularize and become sperm. As adults, nuclei entering the proximal end of each gonad arm cellularize and become oocytes. Not much is known about how germ-cell nuclei progress within the syncytium as they mature from the distal tip to the proximal gonad.

Vesicle fusion is required to deliver new membranes to the point of scission at cytokinesis (Finger and White 2002) and may also be required for maintaining the complex architecture of the gonad. RNAi depletion of proteins required for vesicle fusion could hinder the restructuring of membrane barriers, causing it to occur less efficiently and impeding the normal development of germ cells. In wild-type animals, a less-efficient membrane trafficking system might be tolerated because the machinery that allows the nuclei to move through the gonad is still operational. But in a *mel-28* mutant, perhaps the germ-cell nuclei also cannot be placed efficiently within the gonad because the nuclear envelope does not have its normal constitution. The combination of an inefficient membrane restructuring system and an inefficient nuclear placement system could lead to aberrant formation of the germ cells and thus sterility. If this phenomenon explains the genetic interaction we observe, then we might expect to observe gross architectural problems with the germline, in which the nuclei are not properly partitioned.

An alternative explanation is that the nuclear envelopes in the distal gonad do not reform properly after mitosis. Vesicle transport and membrane rearrangement also are important for the rebuilding of the nuclear envelope after mitosis (Jantsch-Plunger and Glotzer 1999). In vertebrate systems, MEL-28/ELYS is required to efficiently recruit the Nup107-160 subcomplex to the reforming nuclear pore early in postmitotic nuclear envelope reformation (Walther *et al.* 2003; Franz *et al.* 2007; Rasala *et al.* 2008). This step occurs before the recruitment of integral membrane nucleoporins to the reforming nuclear envelope. If the early steps of rebuilding the pore are crippled by the *mel-28* mutation and the later steps of adding membrane to the reforming nuclear envelope are hindered by the disruptions to vesicle trafficking, then perhaps the nuclei at the distal tip cannot form properly functioning nuclear envelopes.

### Nuclear envelope

MEL-28/ELYS is needed in rapidly dividing cells. In *C. elegans*, early embryonic cells divide about once every 10 min, which is much faster than cell division rates during later development. These quick cell divisions require the efficient rebuilding of the nuclear pore. This may explain why *mel-28* mutants survive to adulthood as long as they are rescued by a maternal contribution: maternally provided MEL-28 could allow those rapid early divisions, whereas the slower divisions of later development do not require MEL-28. In the zebrafish *flotte lotte* mutant, which carries a mutation in the *mel-28/elys* gene, proliferative tissues such as the intestine and the optic tectum are most severely affected (Davuluri *et al.* 2008). Because the germline is the most proliferative tissue in *C. elegans* postembryonic development, it might be expected that depletion of genes that work together with *mel-28* to support the nuclear pore would cause sterility in *mel-28* animals.

Just four of the 21 nucleoporin genes we tested caused sterility in N2 animals, suggesting that the nuclear pore is somewhat robust to perturbation in adult cells. The genetic interactions we observed between the *npp* genes and *mel-28* might be explained by this robustness; loss of MEL-28 does not destabilize the pore, but loss of MEL-28 along with an additional component does. Not all nuclear pore components caused a synthetic phenotype with *mel-28*. Even within the Nup107-160 scaffold, we identified just four of the eight genes as *mel-28* genetic interactors. This could indicate that not all nucleoporins contribute equally to the robustness of the nuclear pore structure. Indeed, other studies have shown that individual RNAi-depletion of different members of the Nup107-160 subcomplex does not cause the same phenotype (Galy *et al.* 2003), indicating that even though these proteins are part of the same subcomplex their contributions to the pore are distinct.

### Dynein/dynactin

Dynein is a minus-end directed molecular motor that ferries multiple cellular cargoes, including the nucleus, and dynactin is a regulator of dynein required for its attachment to cargo. Dynein is associated with the nuclear envelope in several different situations. There is evidence from mammalian cell lines that dynein initiates nuclear envelope breakdown by disrupting nuclear membranes associated with centrosome microtubules (Beaudouin *et al.* 2002; Salina *et al.* 2002). Mammalian cell work also revealed a role or dynein in the control of centrosome separation (Busson *et al.* 1998). The dynein-nuclear envelope connection in mammalian cells is mediated by three nonredundant molecular linkages and is required both for the attachment of the centrosomes to the nucleus and for their proper separation during prophase (Splinter *et al.* 2010; Bolhy *et al.* 2011; Jodoin *et al.* 2012).

In the *C. elegans* embryo, the centrosome attaches to the nuclear envelope via ZYG-12, a Hook domain protein that connects with SUN-1 within the nuclear envelope. This attachment is required for the dynein-mediated movement of the pronuclei (Gonczy *et al.* 1999; Yoder and Han 2001; Malone *et al.* 2003). Within the *C. elegans* gonad, the ZYG-12-dynein connection is necessary for the proper positioning of the nuclei within the syncytium; inhibition of ZYG-12 binding to dynein causes mispositioning of the nuclei and microtubule disarray (Zhou *et al.* 2009). Dynactin has also been found at the nuclear envelope in *C. elegans* embryos and in prophase I germline nuclei (Terasawa *et al.* 2010).

In *mel-28* mutant embryos, the nuclear envelope shows severe abnormalities and the centrosomes are detached from the male pronucleus (Fernandez and Piano 2006). One explanation for this centrosome detachment phenotype could be that in the mutant embryos the nuclear envelopes do not have ZYG-12. If the nuclear envelopes within the *mel-28* mutant gonad also had compromised ZYG-12, the gonad might be sensitive to dynein levels. Thus when dynein levels are also lowered, there is no longer enough tension to keep the nuclei in place at the periphery of the gonad, leading to germline defects and a failure to produce a normal brood size.

In summary, we have screened ~85% of the *C. elegans* genome using RNAi and found 65 genes that cause sterility in *mel-28* animals but not the N2. This list of genes includes those that encode components of the nuclear pore, which is consistent with the well-studied function of MEL-28/ELYS in postmitotic rebuilding of the nuclear pore and which validates our screen. In addition, we identified genes implicated in the proteasome, dynein function, vesicle transport, and cell matrix attachments. Some of these categories were unexpected, and reveal connections between seemingly distinct cellular processes that collaborate with *mel-28* during postembryonic development.

## Supplementary Material

Supporting Information
