# Supplementary material for: Uncovering Buffered Pleiotropy: A Genome-Scale Screen for mel-28 Genetic Interactors in Caenorhabditis elegans
Source: G3 (Bethesda). 2013 Nov 26;4(1):185–96. doi: 10.1534/g3.113.008532 (PMC3887534; doi:10.1534/g3.113.008532)
Supplement: Supporting Information [file supp_4_1_185__index.html]

Uncovering Buffered Pleiotropy: A Genome-Scale Screen for mel-28 Genetic Interactors in Caenorhabditis elegans — Supporting Information 

# Uncovering Buffered Pleiotropy: A Genome-Scale Screen for *mel-28* Genetic Interactors in *Caenorhabditis elegans*

## Supporting Information for Fernandez *et al.*, 2014

**Files in this Data Supplement:**

- Supporting Information - Figure S1 and Tables S1-S2 (PDF, 8 MB)
- Figure S1 - *mel-28* genetic interactor phenotypes (PDF, 8 MB)
- Table S1 - Brood size data for *mel-28* genetic interactors (PDF, 513 KB)
- Table S2 - GO-enriched terms amongst *mel-28* genetic interactors (PDF, 511 KB)
