# Supplementary material for: Uncovering Buffered Pleiotropy: A Genome-Scale Screen for mel-28 Genetic Interactors in Caenorhabditis elegans
Source: G3 (Bethesda). 2013 Nov 26;4(1):185–96. doi: 10.1534/g3.113.008532 (PMC3887534; doi:10.1534/g3.113.008532)
Supplement: Supporting Information [file supp_g3.113.008532_008532SI.pdf]

G3: Genes | Genomes | Genetics

Uncovering Buffered Pleiotropy: A Genome-Scale Screen for *mel-28* Genetic Interactors in *Caenorhabditis elegans*

Anita G. Fernandez[*], Emily K. Mis[§], Allison Lai[*], Michael Mauro[*], Angela Quental[*], Carly Bock[*], and Fabio Piano[§#]

[*] Fairfield University Biology Department, Fairfield, CT, 06824
[§] New York University Department of Biology and Center for Genomics and Systems Biology, New York, NY 10003
[#] New York University, Abu Dhabi, United Arab Emirates

DOI: 10.1534/g3.113.008532

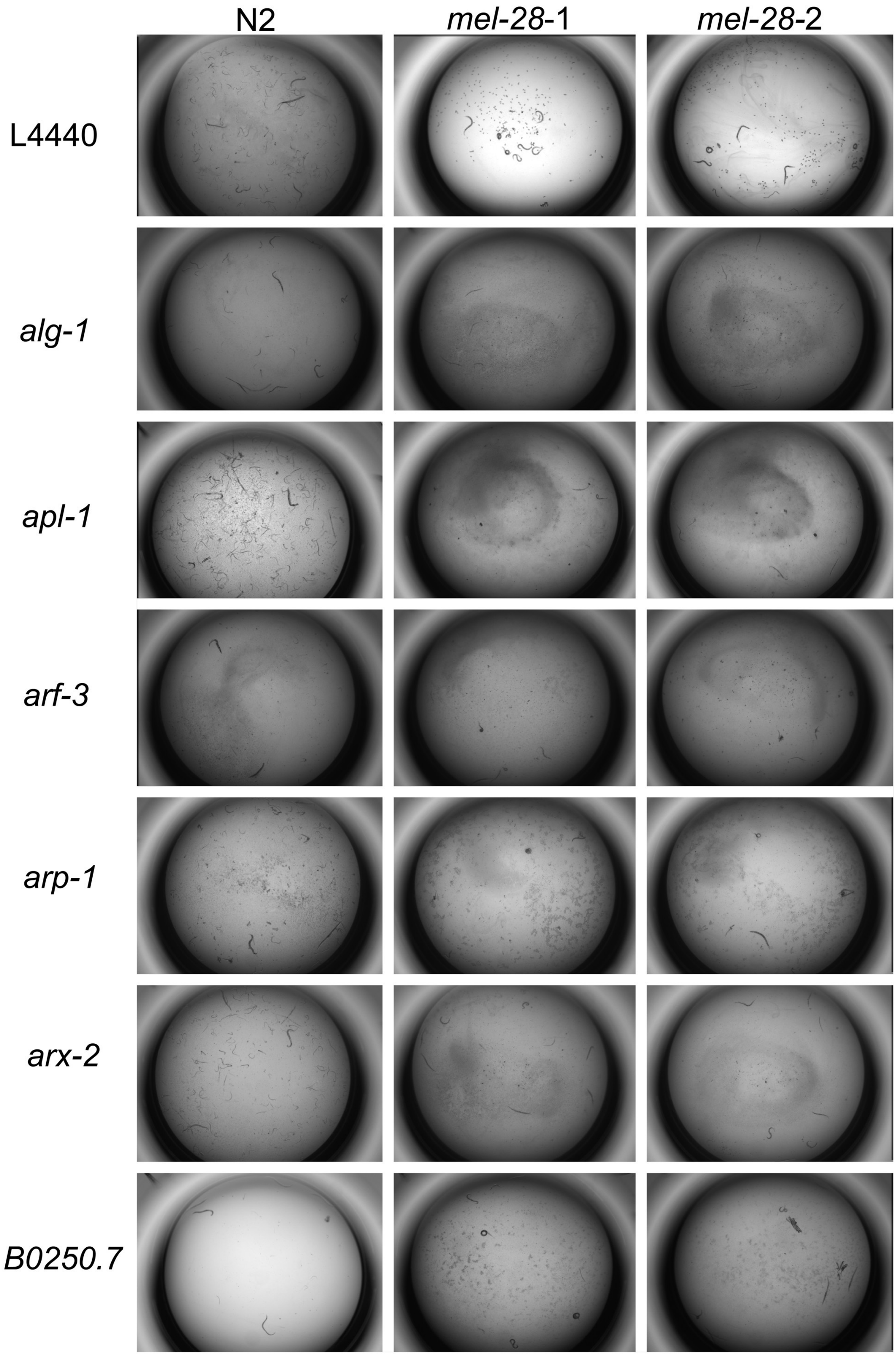
N2
mel-28-1
mel-28-2
L4440
alg-1
apl-1
arf-3
arp-1
arx-2
B0250.7

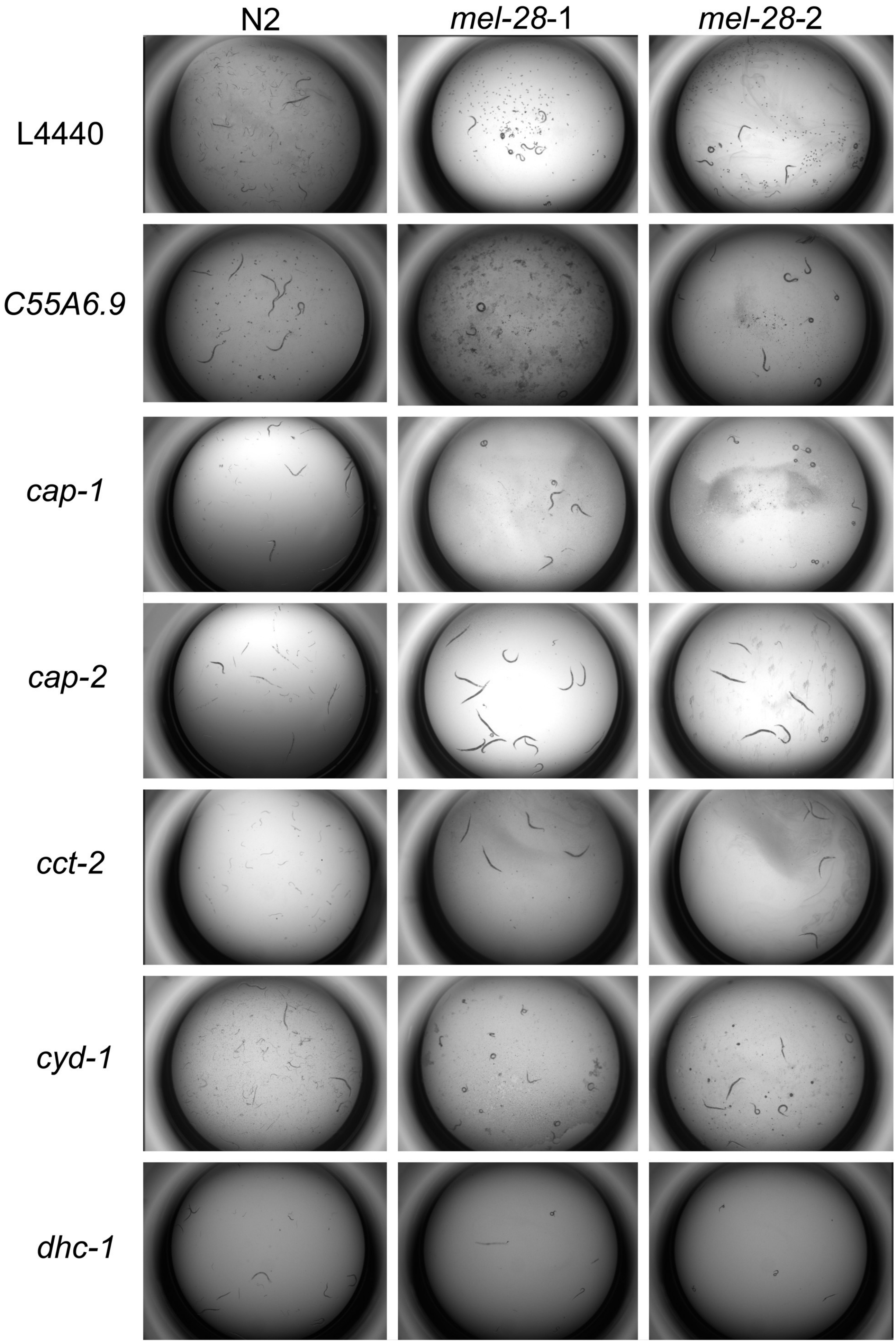
N2
mel-28-1
mel-28-2
L4440
C55A6.9
cap-1
cap-2
cct-2
cyd-1
dhc-1

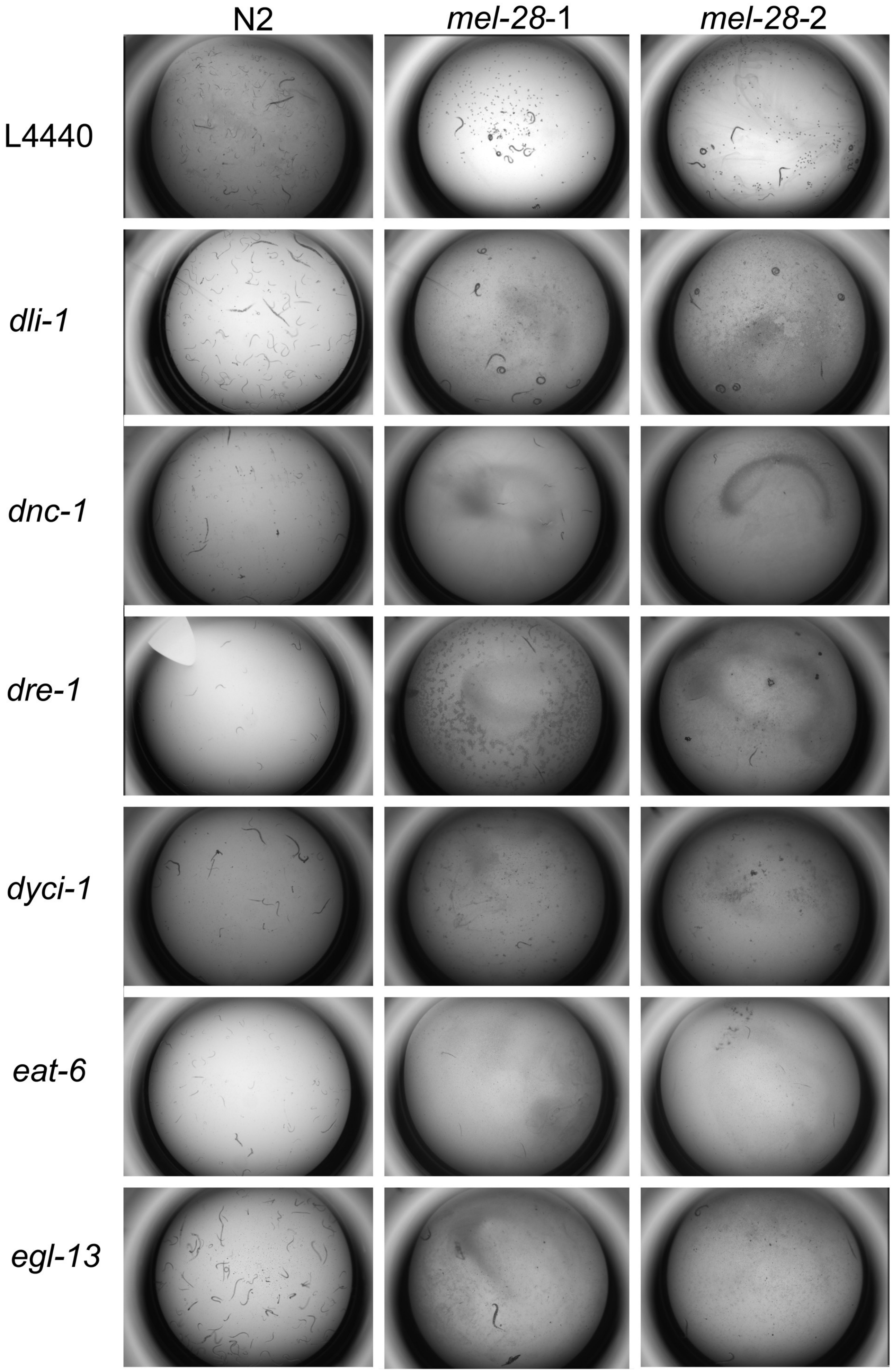

N2
mel-28-1
mel-28-2
L4440
dli-1
dnc-1
dre-1
dyci-1
eat-6
egl-13

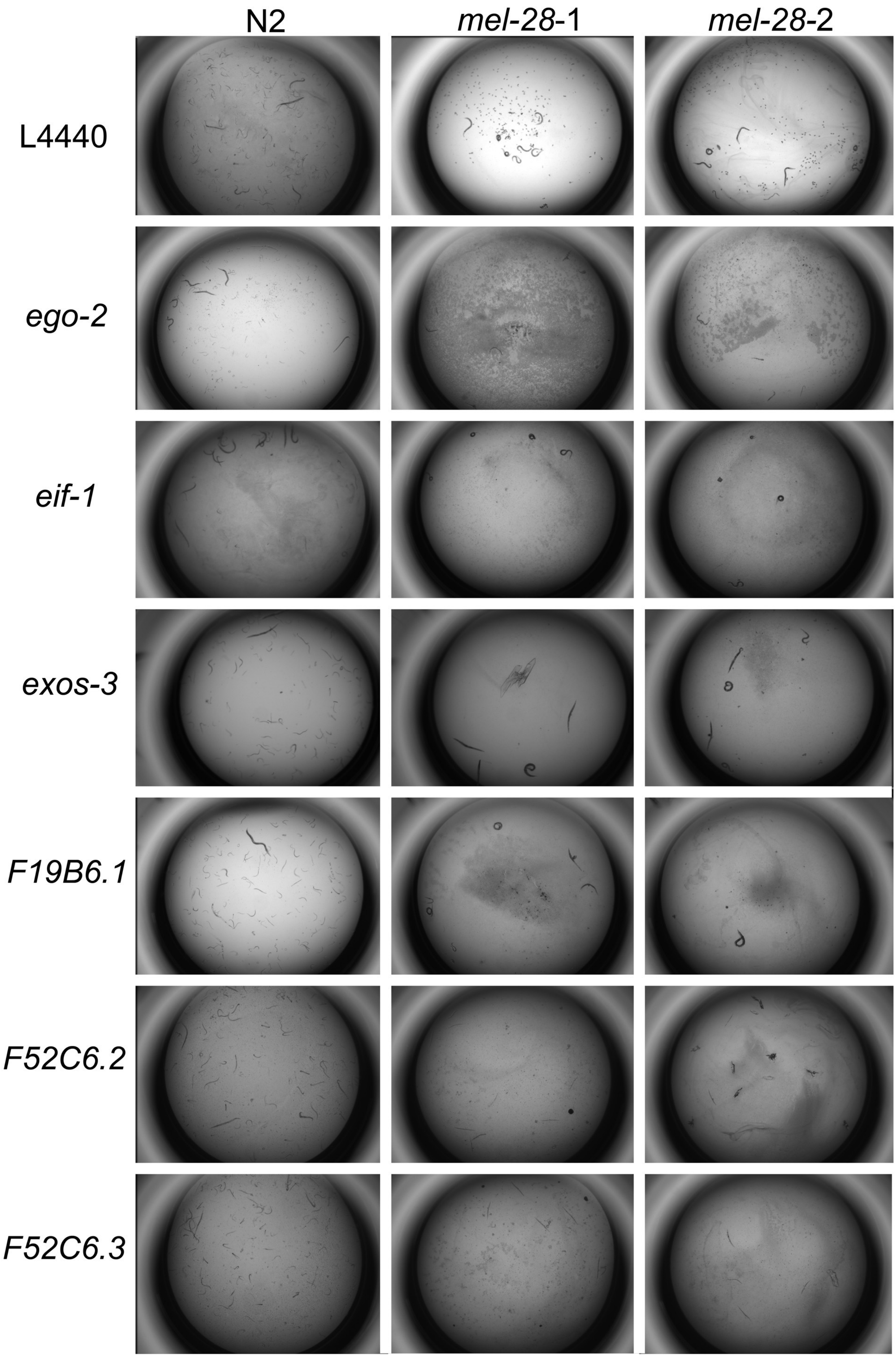

N2
mel-28-1
mel-28-2
L4440
ego-2
eif-1
exos-3
F19B6.1
F52C6.2
F52C6.3

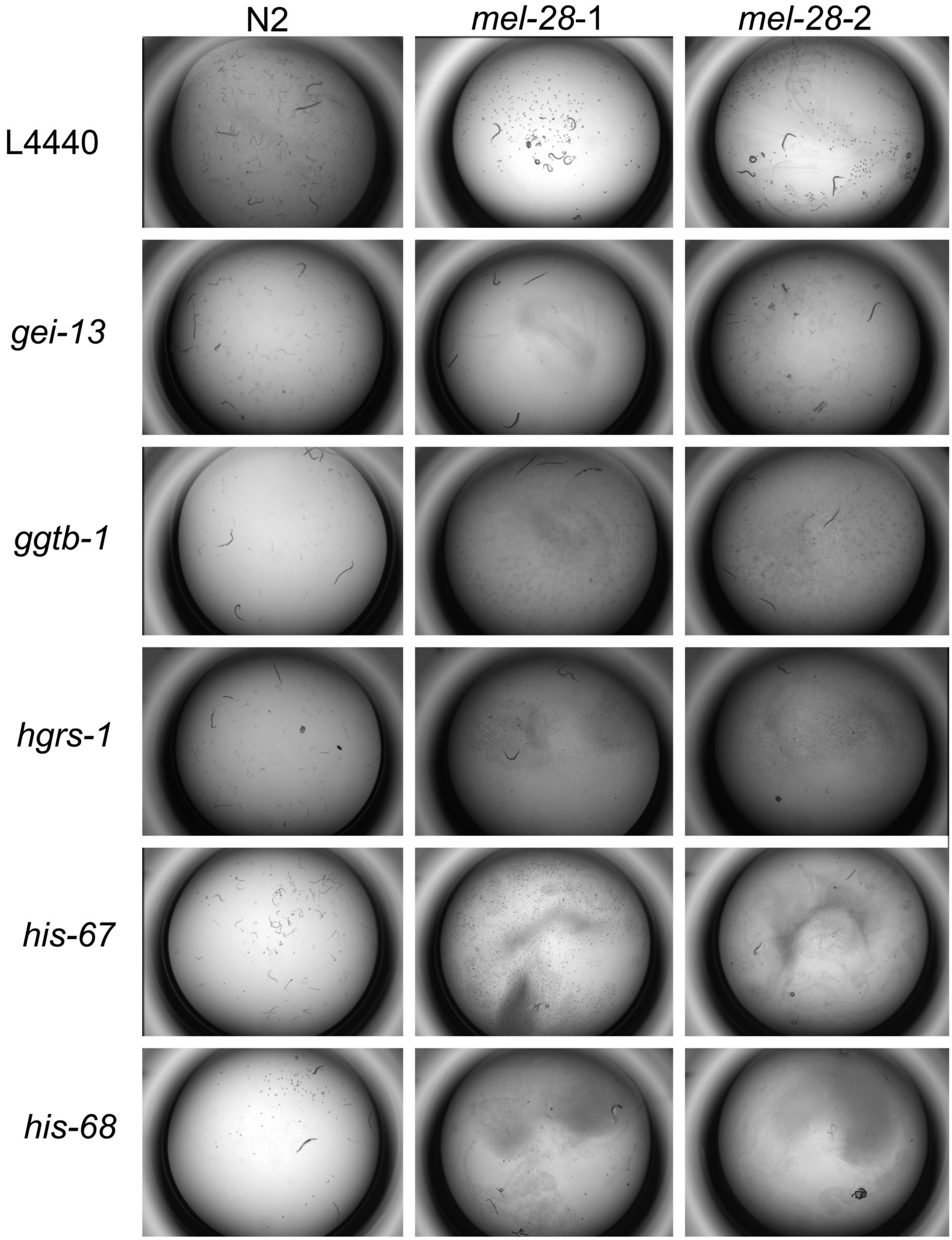

N2
mel-28-1
mel-28-2
L4440
gei-13
ggtb-1
hgrs-1
his-67
his-68

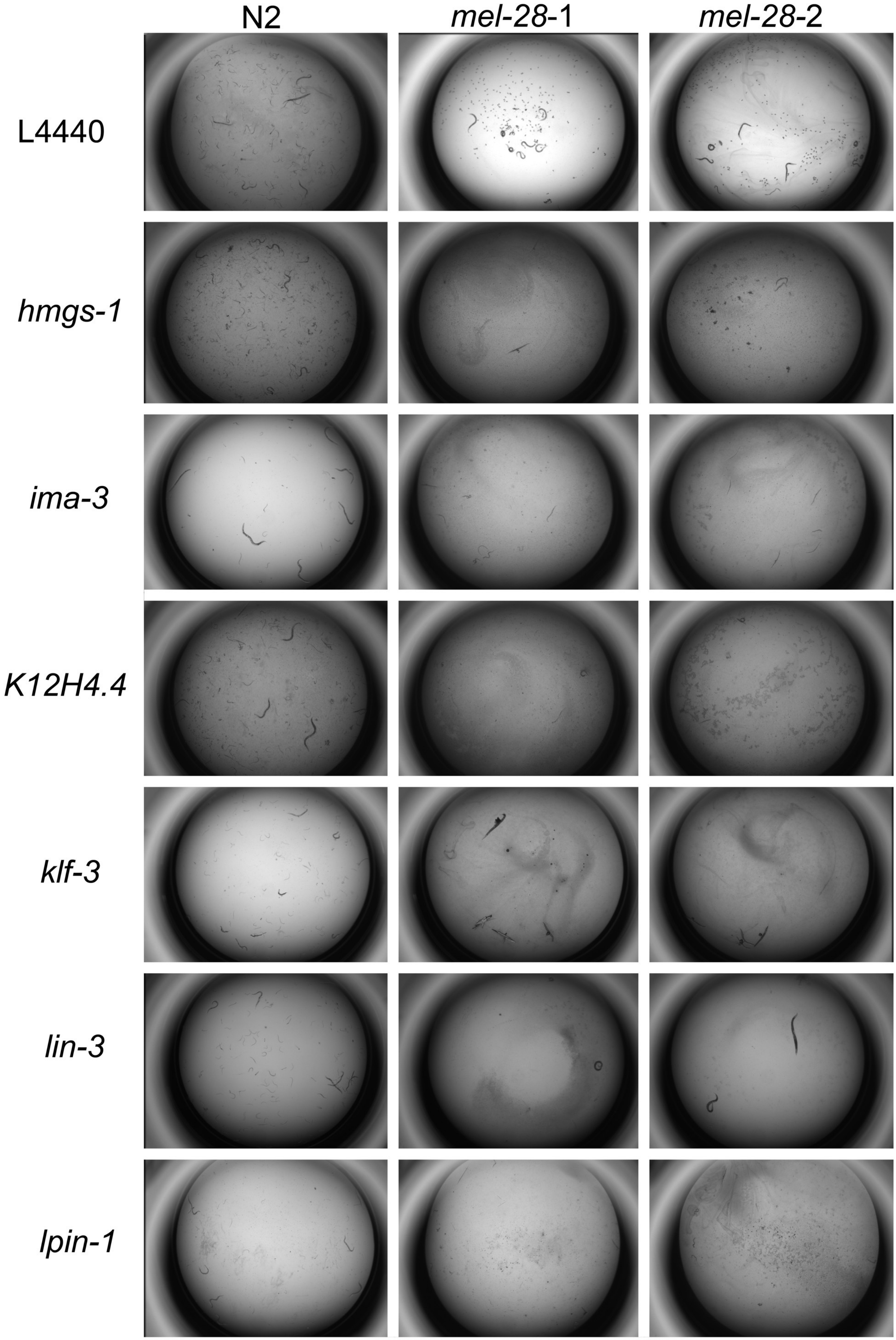
N2
mel-28-1
mel-28-2
L4440
hmgs-1
ima-3
K12H4.4
klf-3
lin-3
lpin-1

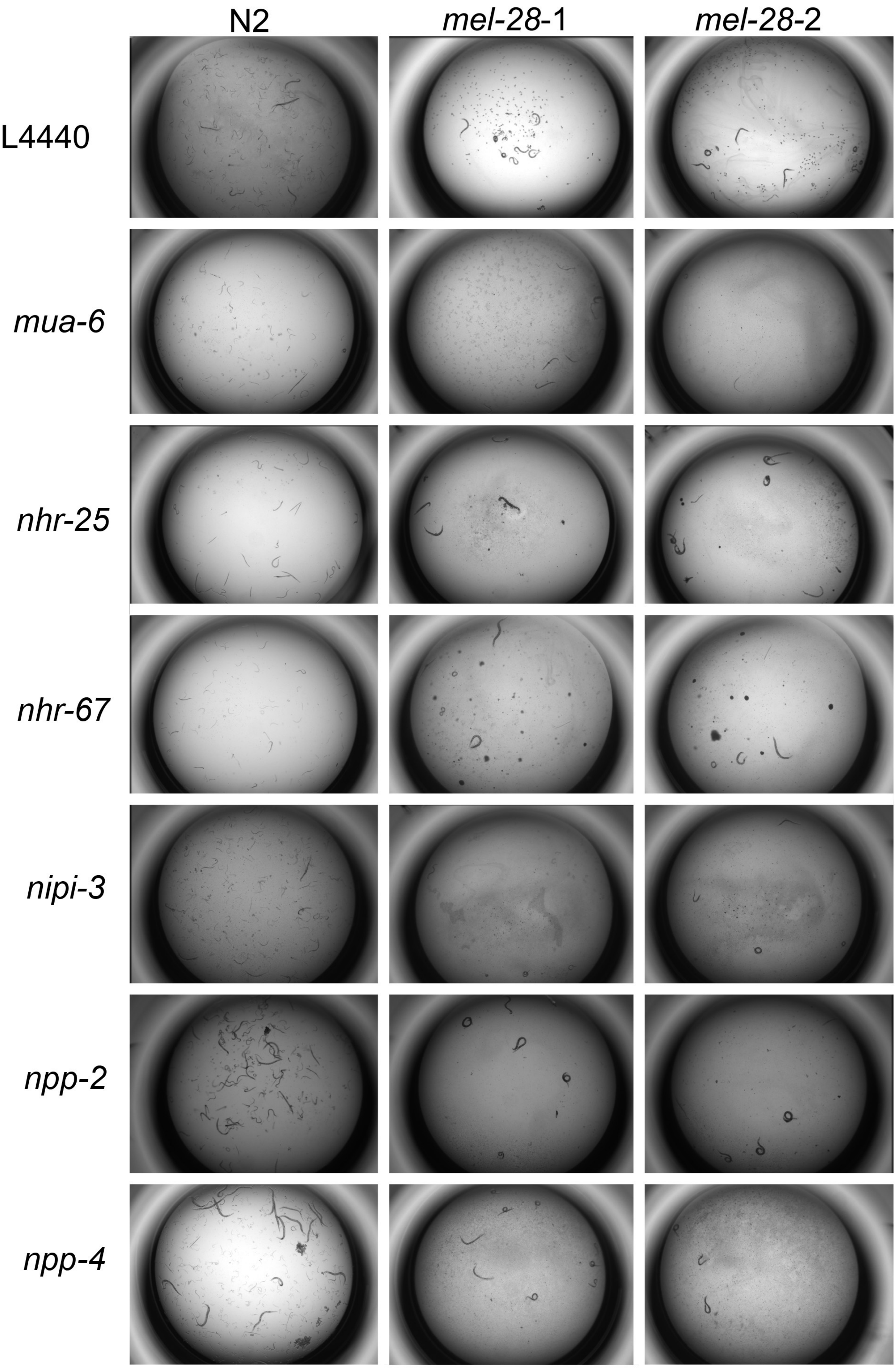
N2
mel-28-1
mel-28-2
L4440
mua-6
nhr-25
nhr-67
nipi-3
npp-2
npp-4

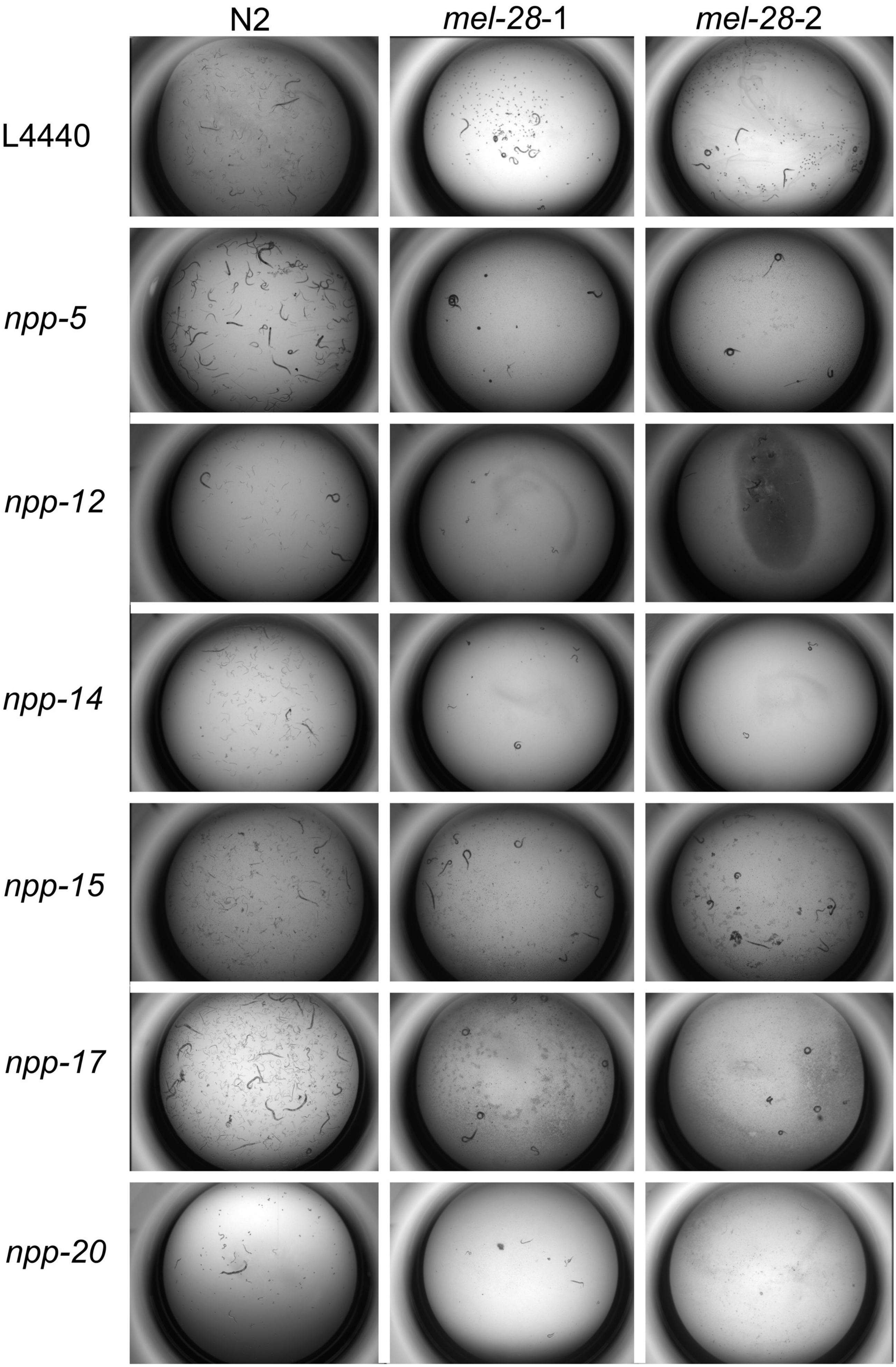
N2
mel-28-1
mel-28-2
L4440
npp-5
npp-12
npp-14
npp-15
npp-17
npp-20

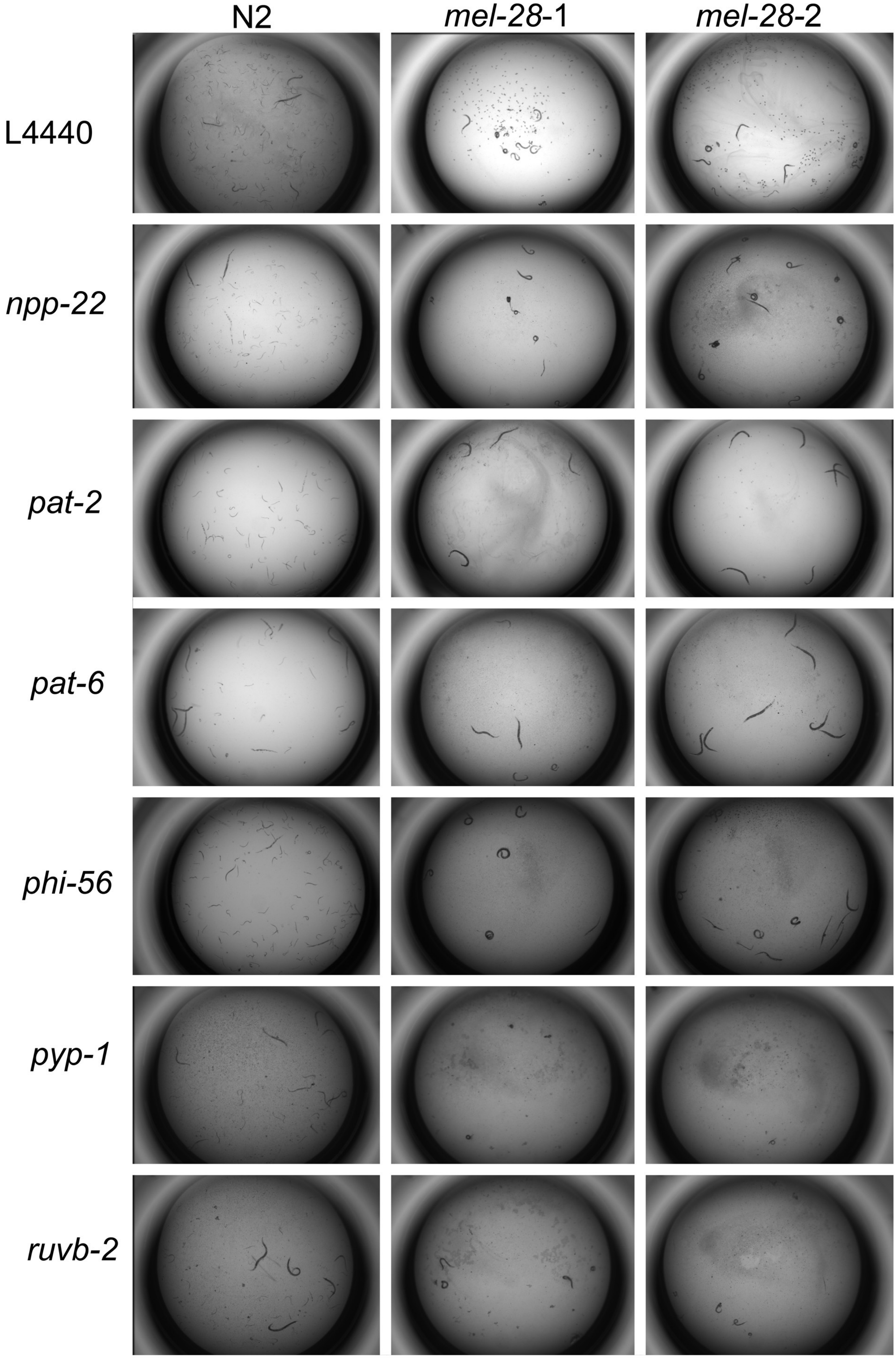

N2
mel-28-1
mel-28-2
L4440
npp-22
pat-2
pat-6
phi-56
pyp-1
ruvb-2

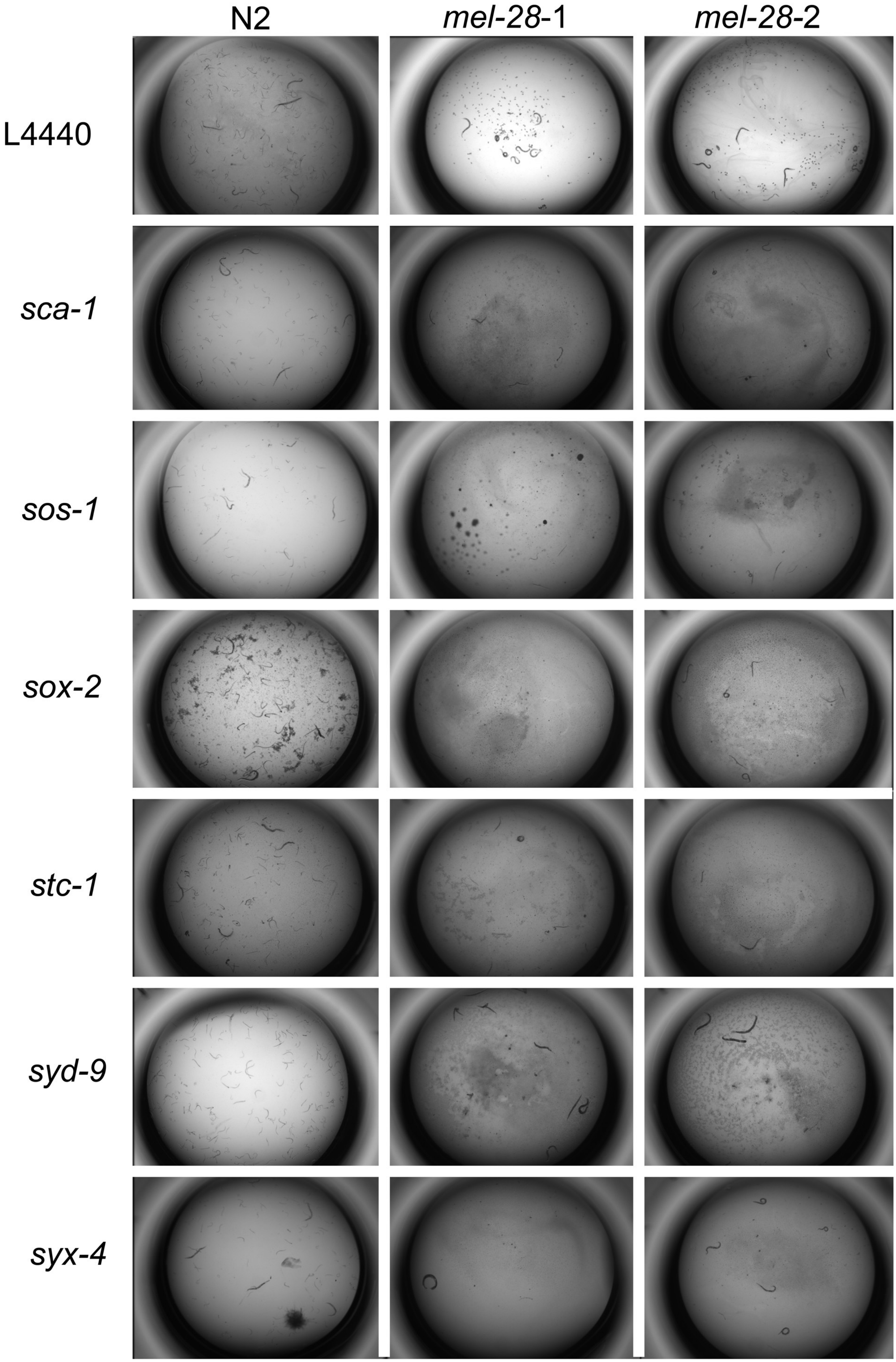
N2
mel-28-1
mel-28-2
L4440
sca-1
sos-1
sox-2
stc-1
syd-9
syx-4

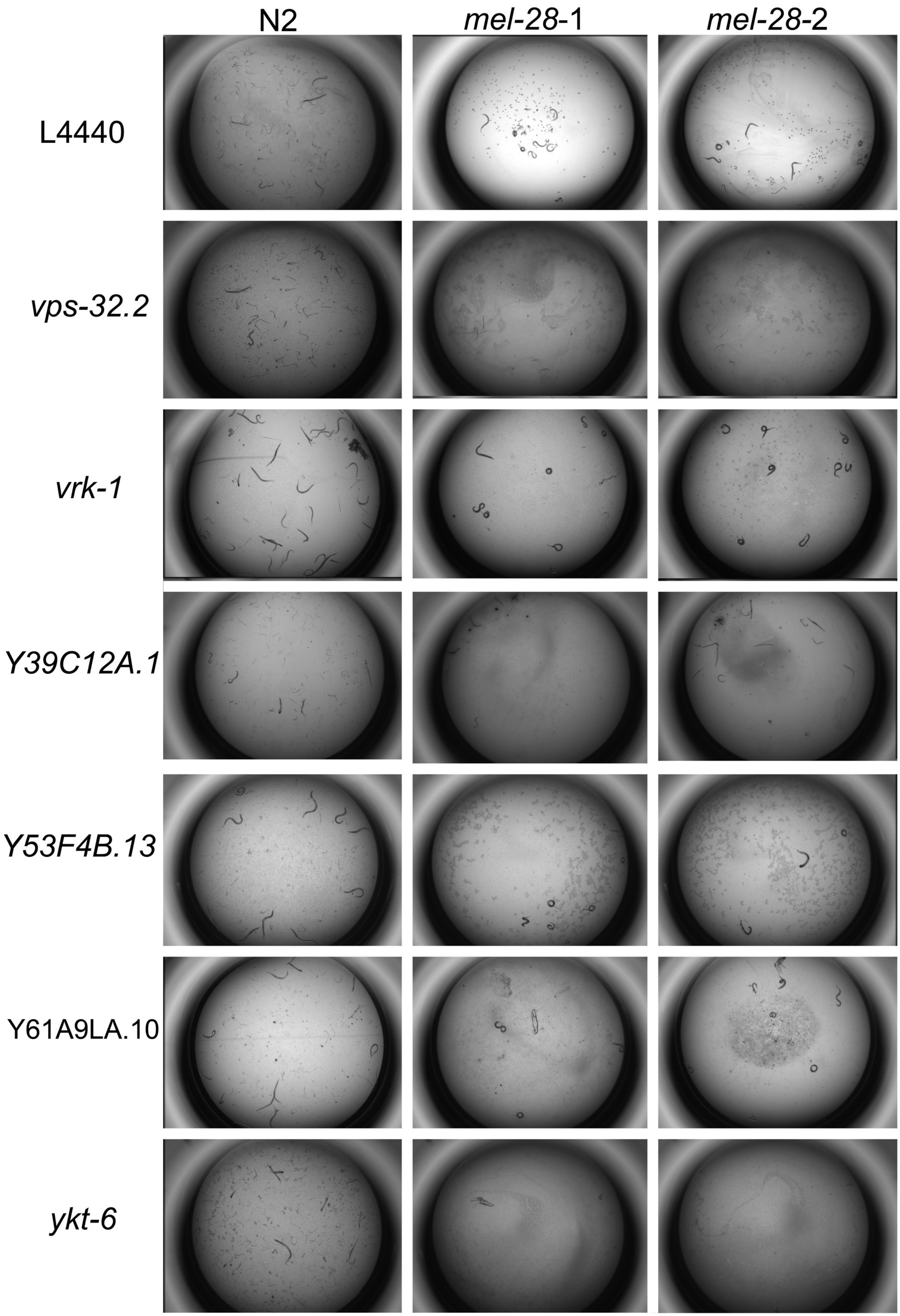

Figure S1 *mel-28* genetic interactor phenotypes

Table S1 Brood size data for *mel-28* genetic interactors

gene	progeny/adult (N2)	eggs/ adult (*mel-28)*	ratio N2 brood size: *mel-28* brood size	genetic interaction
pat-2	25.3	0.8	31.6	synthetic sterility
pat-6	7.3	0.8	9.1	enhancement
klf-3	13.9	0.0	>3	synthetic sterility
his-67	9.9	2.7	3.7	enhancement
his-68	10.2	0.0	>3	enhancement
pyp-1	2.8	0.0	>3	enhancement
ruvb-2	9.5	0.7	13.6	enhancement
dhc-1	1.7	0.0	>3	enhancement
dyci-1	4	0.0	>3	enhancement
dli-1	16	0.4	40	synthetic sterility
dnc-1	14.1	0.6	23.5	synthetic sterility
cap-1	12	0.6	20	enhancement
cap-2	8.4	1.4	6	enhancement
arp-1	16.7	1.8	9.3	synthetic sterility
npp-2	8.7	0.7	12.4	enhancement
npp-4	14.7	0.0	>3	synthetic sterility
npp-5	18.2	1.8	10.1	synthetic sterility
npp-12	21.3	0.3	71	synthetic sterility
npp-14	16.2	0.0	>3	synthetic sterility
npp-15	16	0.0	>3	synthetic sterility
npp-17	12.3	0.3	41	enhancement
npp-20	8.5	0.0	>3	enhancement
npp-22	25	0.0	>3	synthetic sterility
ima-3	6	0.0	>3	enhancement
vrk-1	4	1.1	3.6	enhancement
lpin-1	5	0.0	>3	enhancement
cct-2	17.3	0.2	86.5	synthetic sterility
stc-1	13.6	1.3	10.5	synthetic sterility
sca-1	13.2	0.0	>3	synthetic sterility
phi-56	12.3	0.0	>3	enhancement
K12H4.4	11.8	0.0	>3	enhancement
ggtb-1	2.6	0.0	>3	enhancement
ykt-6	11.7	1.0	11.7	enhancement
syx-4	24.3	0.4	60.8	synthetic sterility
mua-6	18.5	0.7	26.4	synthetic sterility
syd-9	55	0.2	275	synthetic sterility

arf-3	4.2	0.0	>3	enhancement
hgrs-1	17.8	0.0	>3	synthetic sterility
vps-32.2	11.3	0.0	>3	enhancement
Y61A9LA.10	9.4	0.0	>3	enhancement
eif-1	3.7	0.0	>3	enhancement
Y39C12A.1	20.8	0.7	29.7	synthetic sterility
F52C6.2	16	1.8	8.9	synthetic sterility
F52C6.3	14.7	0.0	>3	synthetic sterility
exos-3	11.2	3.0	3.7	enhancement
alg-1	4.9	0.0	>3	enhancement
cyd-1	27.8	0.0	>3	synthetic sterility
ego-2	15	0.6	25	synthetic sterility
dre-1	9.3	0.0	>3	enhancement
nipi-3	8.1	1.0	8.1	enhancement
hmgs-1	15.7	0.0	>3	synthetic sterility
nhr-25	17.1	0.0	>3	synthetic sterility
nhr-67	23.3	0.0	>3	synthetic sterility
apl-1	32	0.0	>3	synthetic sterility
F19B6.1	15.6	0.0	>3	synthetic sterility
C55A6.9	6.8	0.2	34	enhancement
egl-13	25	0.0	>3	synthetic sterility
sox-2	17	0.0	>3	synthetic sterility
gei-13	15	0.4	37.5	synthetic sterility
Y53F4B.13	3.8	0.0	>3	enhancement
lin-3	26.7	0.7	38.1	synthetic sterility
arx-2	27.2	0.0	>3	synthetic sterility
eat-6	10.9	0.0	>3	enhancement
B0250.7	3.8	1.0	3.8	enhancement
sos-1	7.8	0.0	>3	enhancement

For each strain (N2 or *mel-28*) the number of progeny per adult was tallied. We did this by adding up the total number of mothers and dividing this number by the total number of progeny from at least two RNAi experiments. For the *dhc-1* gene these tallies came from a single RNAi experiment (see methods for details).

Table S2 GO-enriched terms amongst *mel-28* genetic interactors

N	P value	GO attribute ID	GO attribute name
2	2.04E-05	GO:0008290	F-actin capping protein complex
2	6.11E-05	GO:0005787	signal peptidase complex
3	1.06E-05	GO:0005869	dynactin complex
7	2.39E-11	GO:0005643	nuclear pore
3	1.92E-05	GO:0051028	mRNA transport
7	6.97E-11	GO:0046930	pore complex
10	5.02E-13	GO:0005635	nuclear envelope
5	3.34E-06	GO:0006997	nucleus organization
5	2.25E-05	GO:0007338	single fertilization
5	2.73E-05	GO:0009566	fertilization
10	2.10E-08	GO:0031967	organelle envelope
10	2.20E-08	GO:0031975	envelope
12	2.05E-09	GO:0012505	endomembrane system
7	1.07E-05	GO:0051656	establishment of organelle localization
7	1.30E-05	GO:0051640	organelle localization
7	2.82E-05	GO:0032940	secretion by cell
7	3.11E-05	GO:0046903	secretion
8	2.83E-05	GO:0015031	protein transport
11	1.16E-06	GO:0044428	nuclear part
8	3.15E-05	GO:0045184	establishment of protein localization
14	6.34E-08	GO:0051649	establishment of localization in cell
14	1.62E-07	GO:0051641	cellular localization
21	7.91E-10	GO:0043234	protein complex
20	2.37E-09	GO:0006898	receptor-mediated endocytosis
11	8.38E-06	GO:0008104	protein localization
40	9.36E-12	GO:0040007	growth
20	2.91E-08	GO:0006897	endocytosis
21	3.49E-08	GO:0016192	vesicle-mediated transport
42	7.57E-11	GO:0009792	embryo development ending in birth or egg hatching
23	1.75E-08	GO:0044422	organelle part
21	6.33E-08	GO:0044446	intracellular organelle part
21	6.69E-08	GO:0032991	macromolecular complex
42	1.80E-10	GO:0009790	embryo development
33	6.12E-09	GO:0002119	nematode larval development
33	6.24E-09	GO:0002164	larval development
33	7.71E-09	GO:0009791	post-embryonic development

31	1.40E-08	GO:0051234	establishment of localization
30	2.70E-08	GO:0006810	transport
44	3.85E-08	GO:0048856	anatomical structure development
46	7.61E-08	GO:0007275	multicellular organismal development
45	1.52E-07	GO:0044767	single-organism developmental process
24	2.20E-06	GO:0040010	positive regulation of growth rate
24	2.26E-06	GO:0040009	regulation of growth rate
34	2.33E-07	GO:0051179	localization
26	1.56E-06	GO:0040011	locomotion
46	5.04E-07	GO:0032502	developmental process
46	1.46E-06	GO:0044707	single-multicellular organism process
25	6.07E-06	GO:0045927	positive regulation of growth
46	3.83E-06	GO:0032501	multicellular organismal process
19	7.29E-05	GO:0005515	protein binding
25	1.35E-05	GO:0040008	regulation of growth
27	1.21E-05	GO:0048518	positive regulation of biological process
33	5.12E-06	GO:0000003	reproduction
30	5.50E-05	GO:0043229	intracellular organelle
30	5.63E-05	GO:0043226	organelle

We used FuncAssociate (http://llama.mshri.on.ca/funcassociate_client/html/) to determine the GO term enrichment within the list of 65 *mel-28* genetic interactors identified. The background set was the entire *C. elegans* genome.
