# Supplementary figures and images for: Uncovering Buffered Pleiotropy: A Genome-Scale Screen for mel-28 Genetic Interactors in Caenorhabditis elegans
Source: G3 (Bethesda). 2013 Nov 26;4(1):185–96. doi: 10.1534/g3.113.008532 (PMC3887534; doi:10.1534/g3.113.008532)

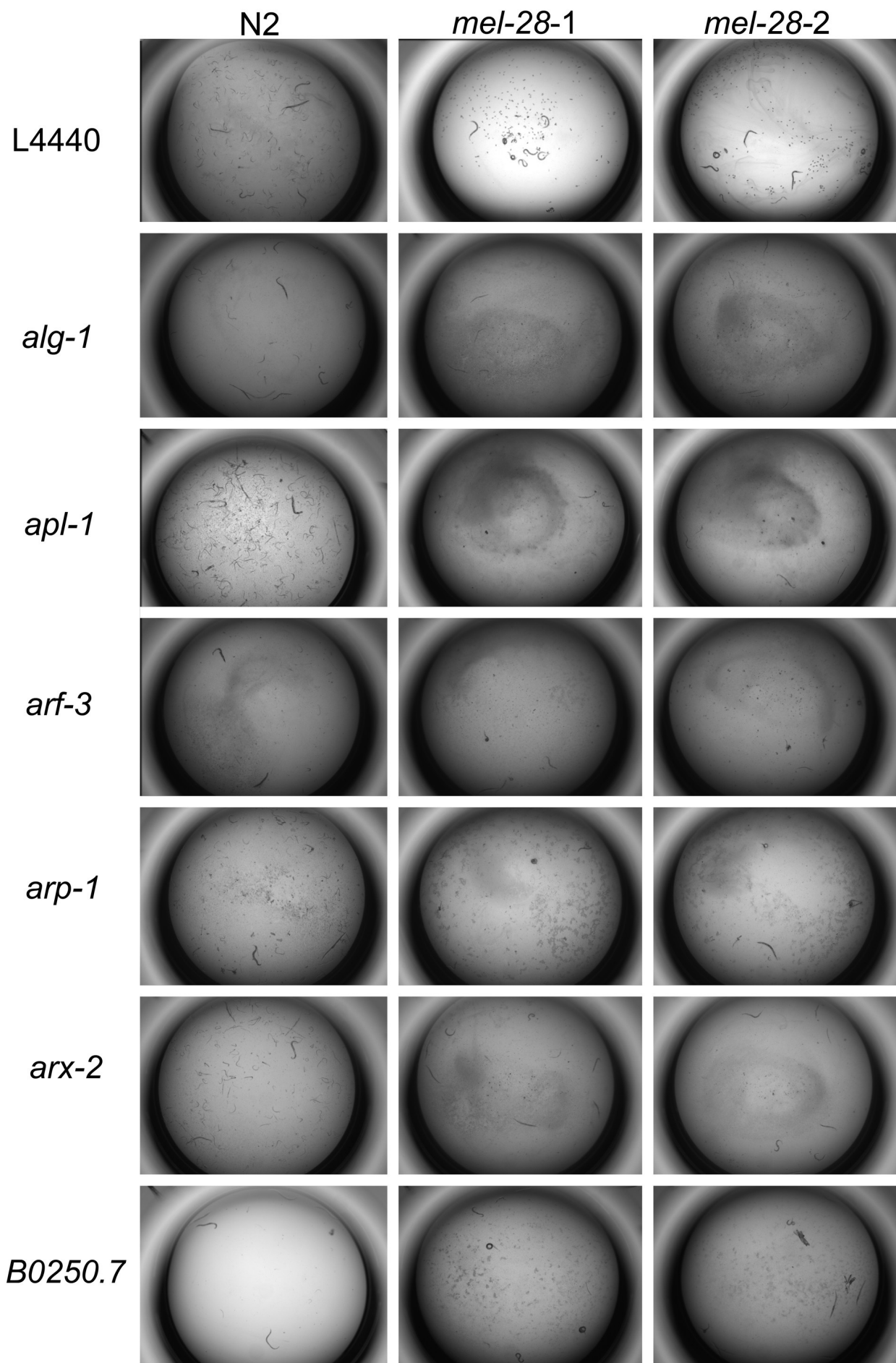

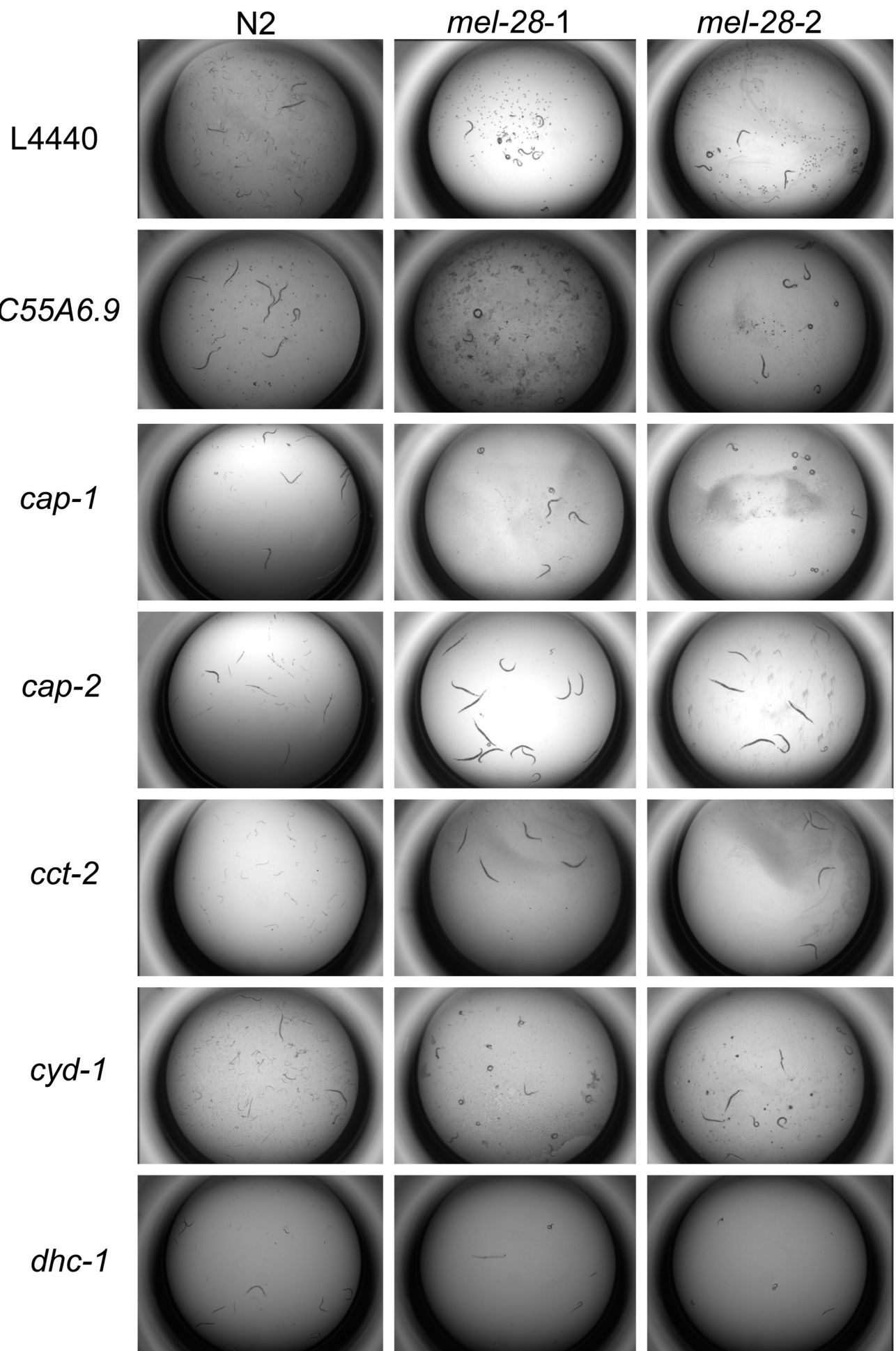

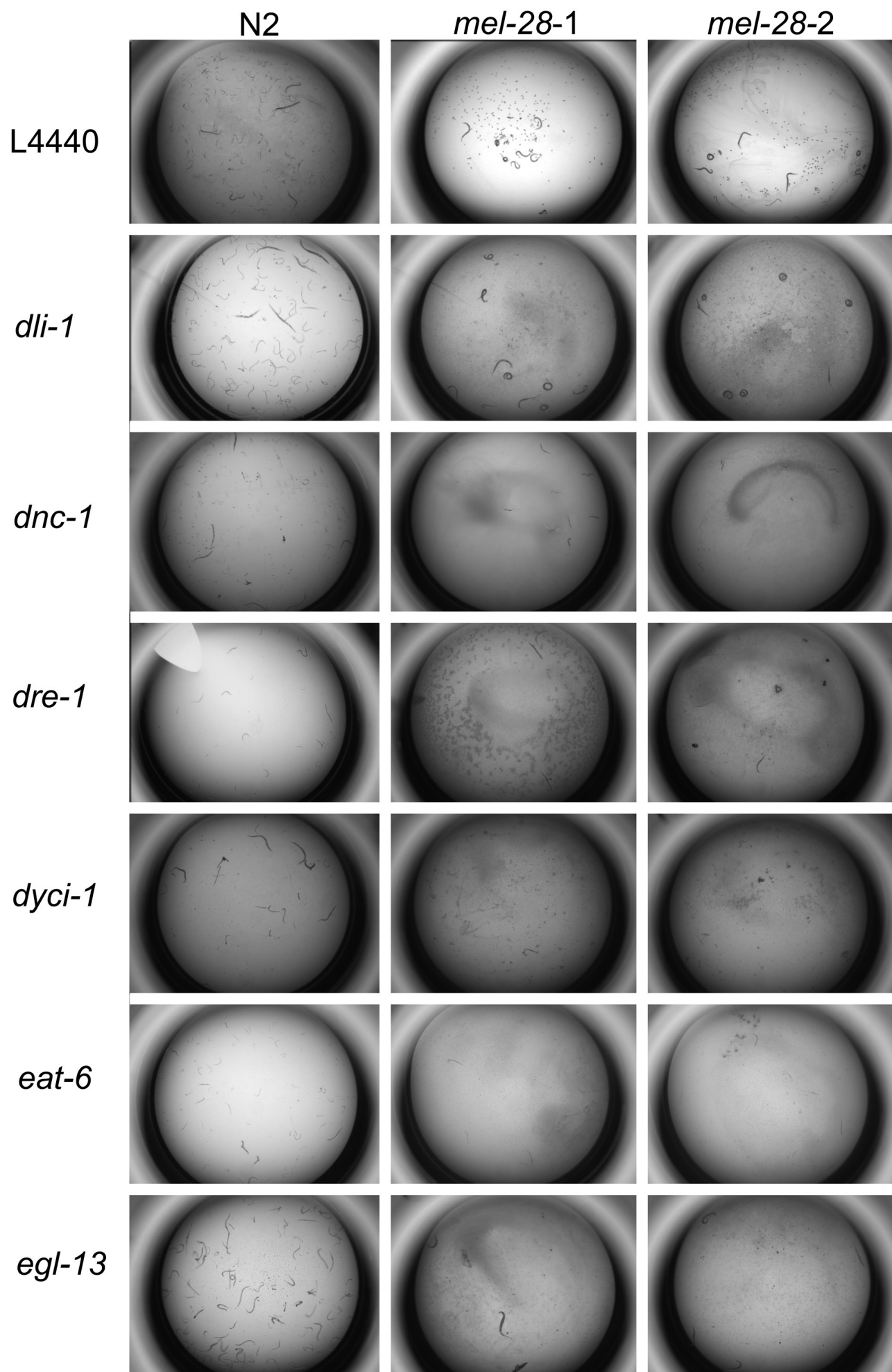

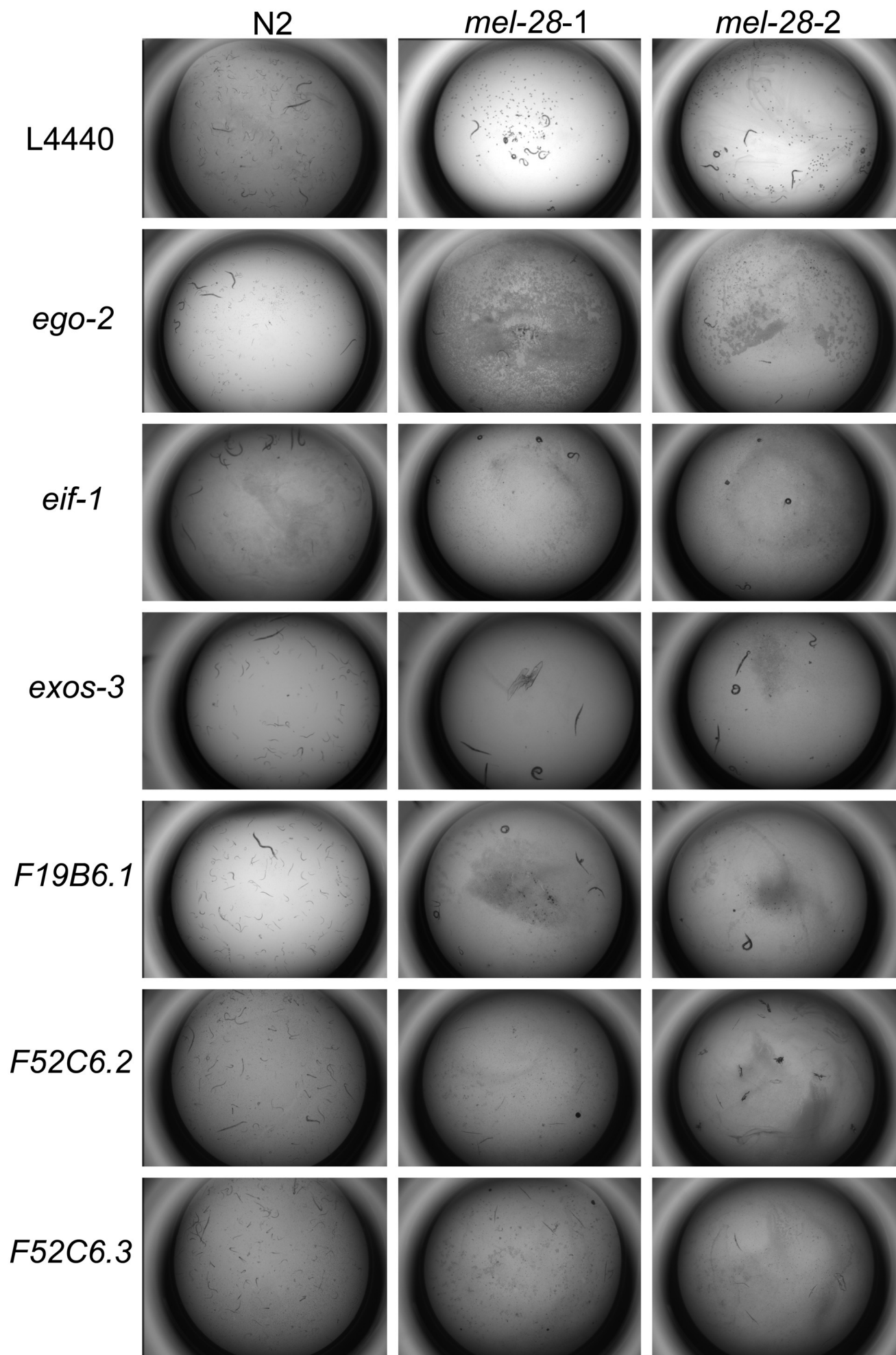

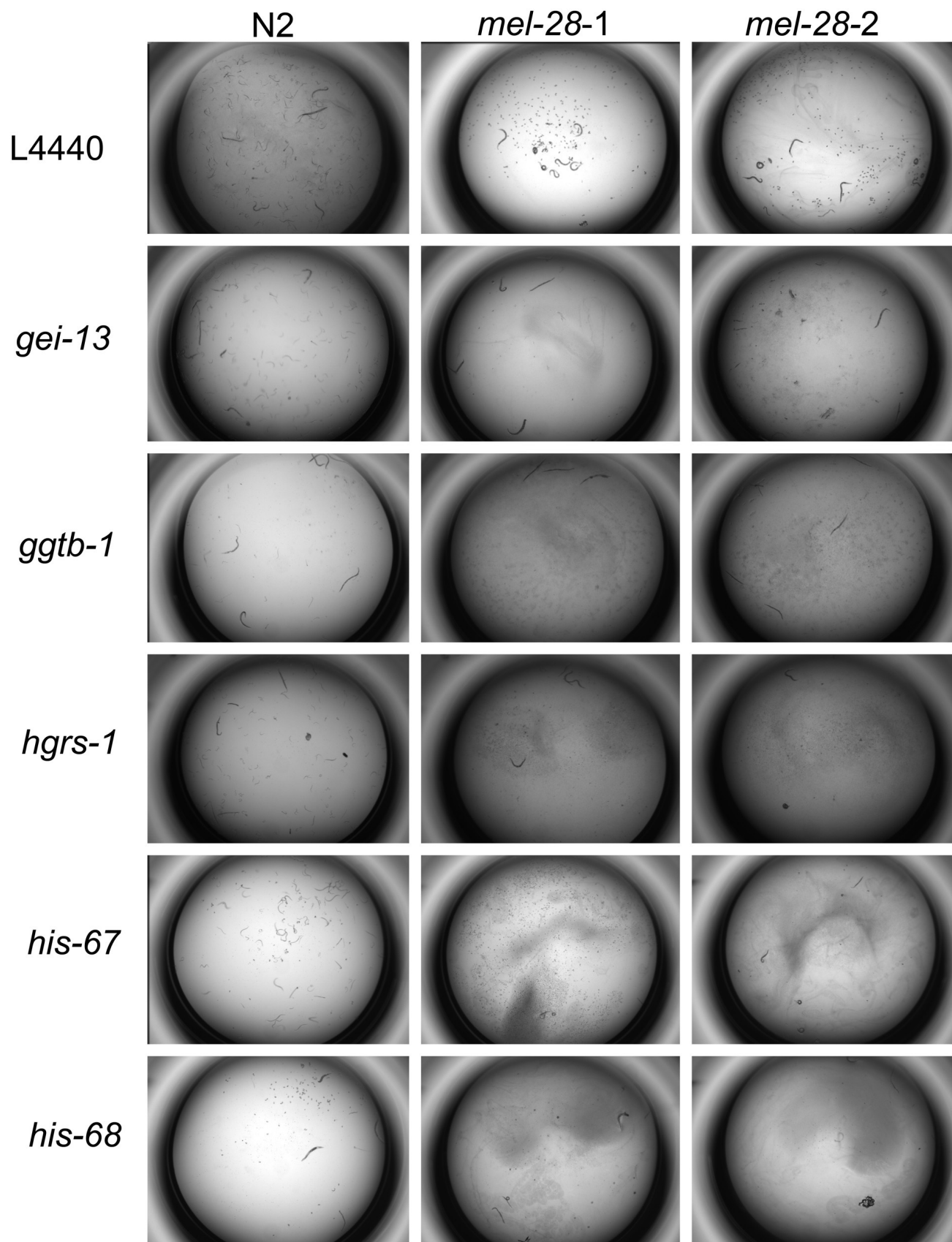

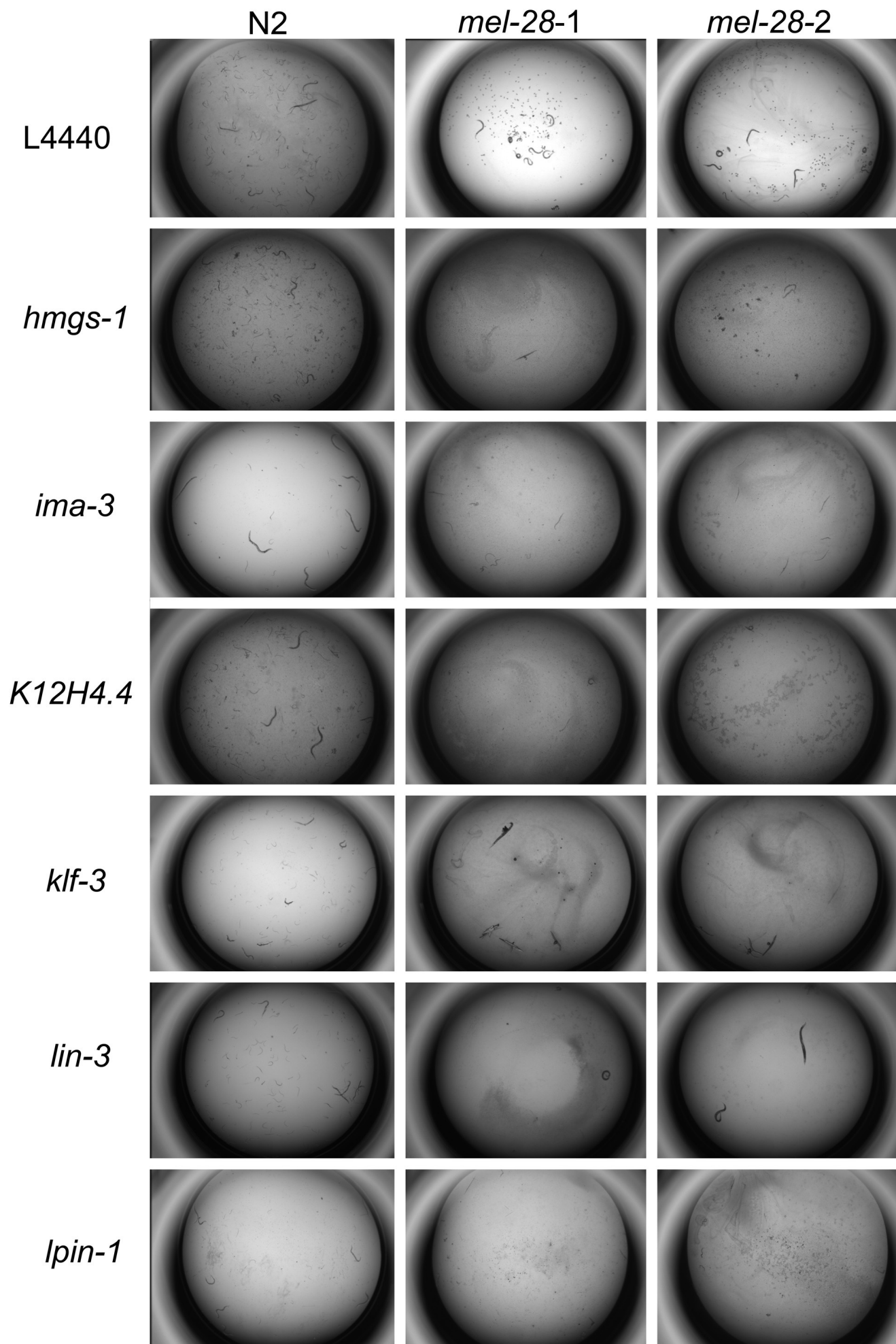

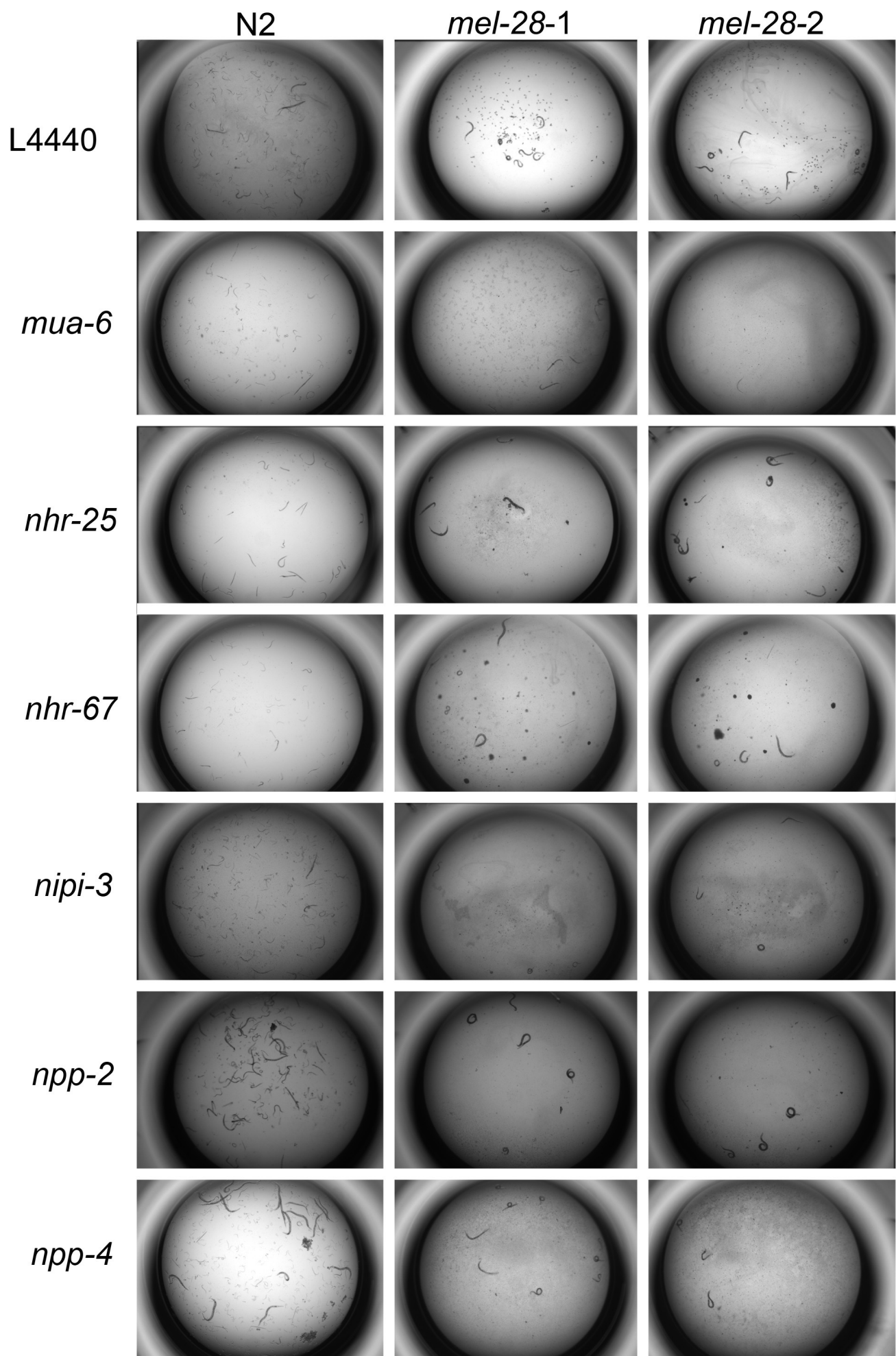

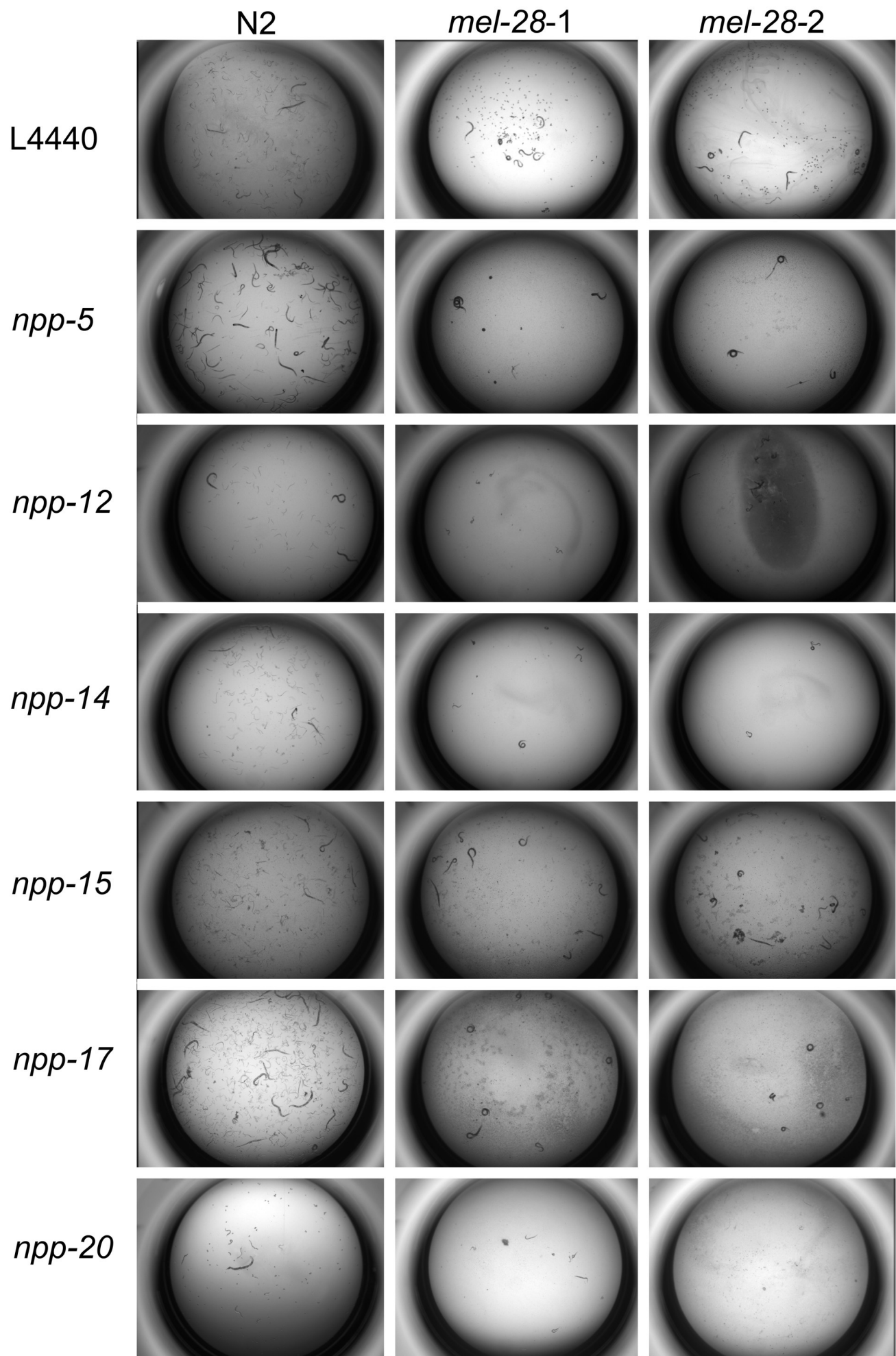

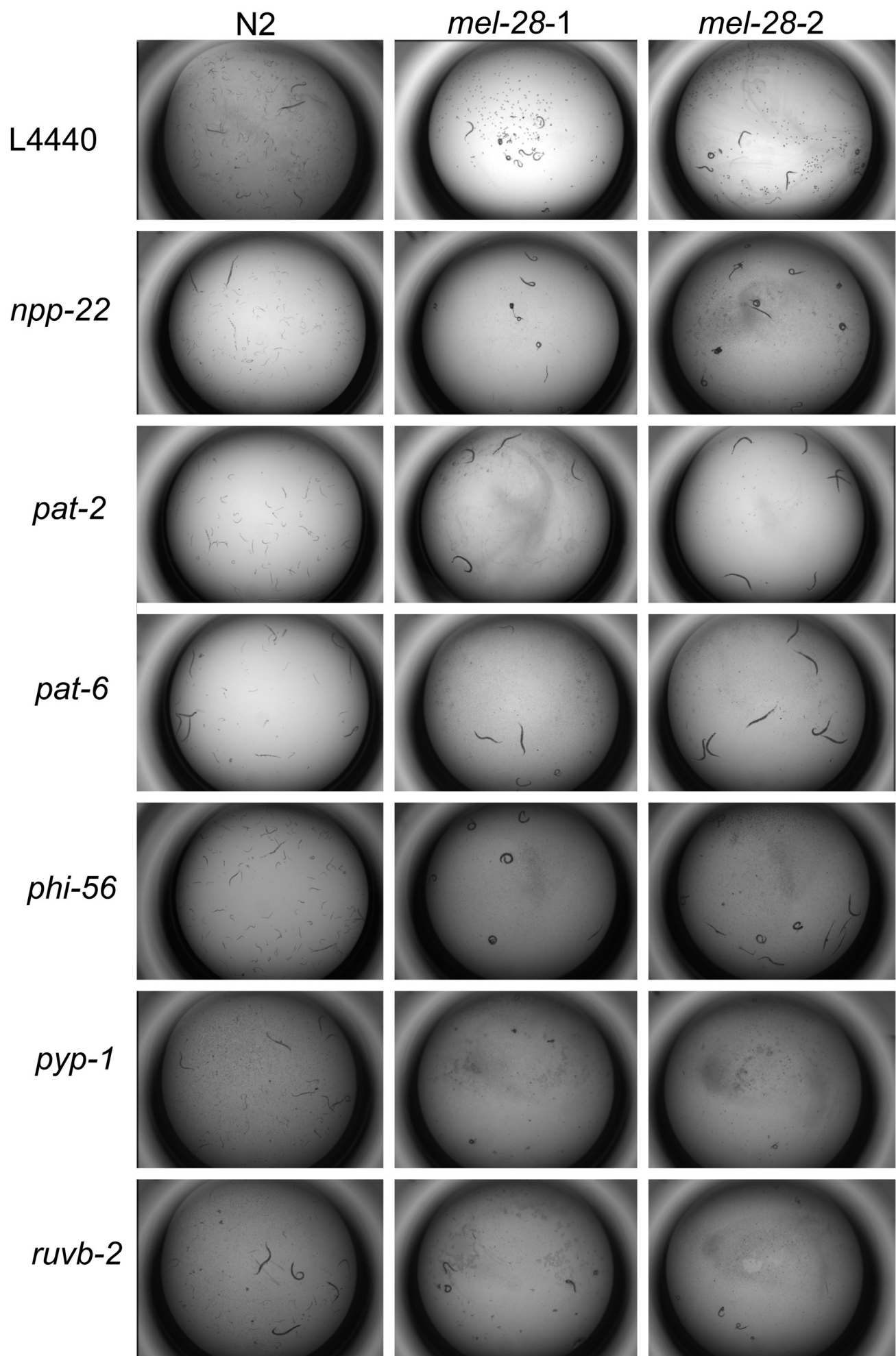

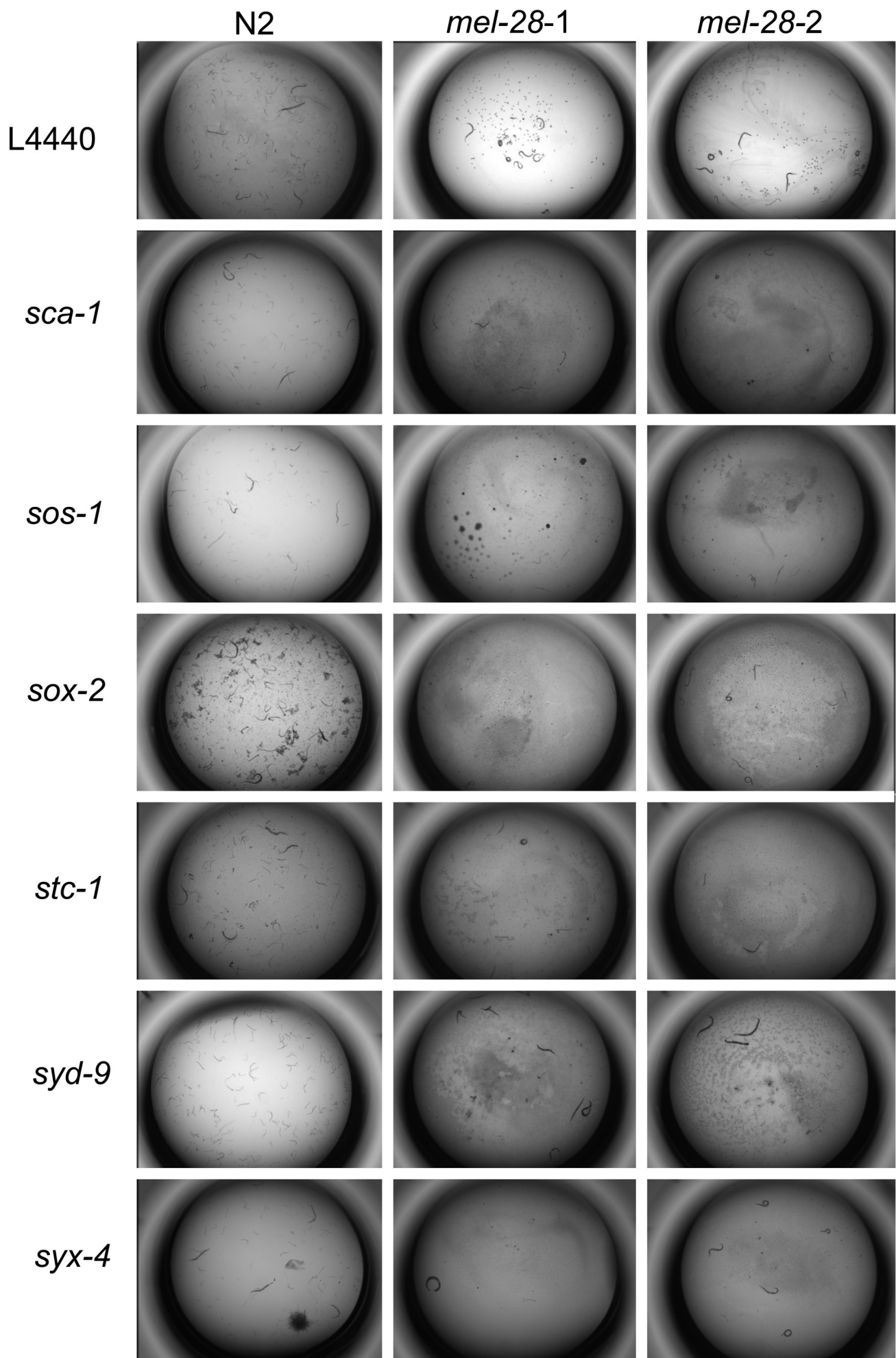

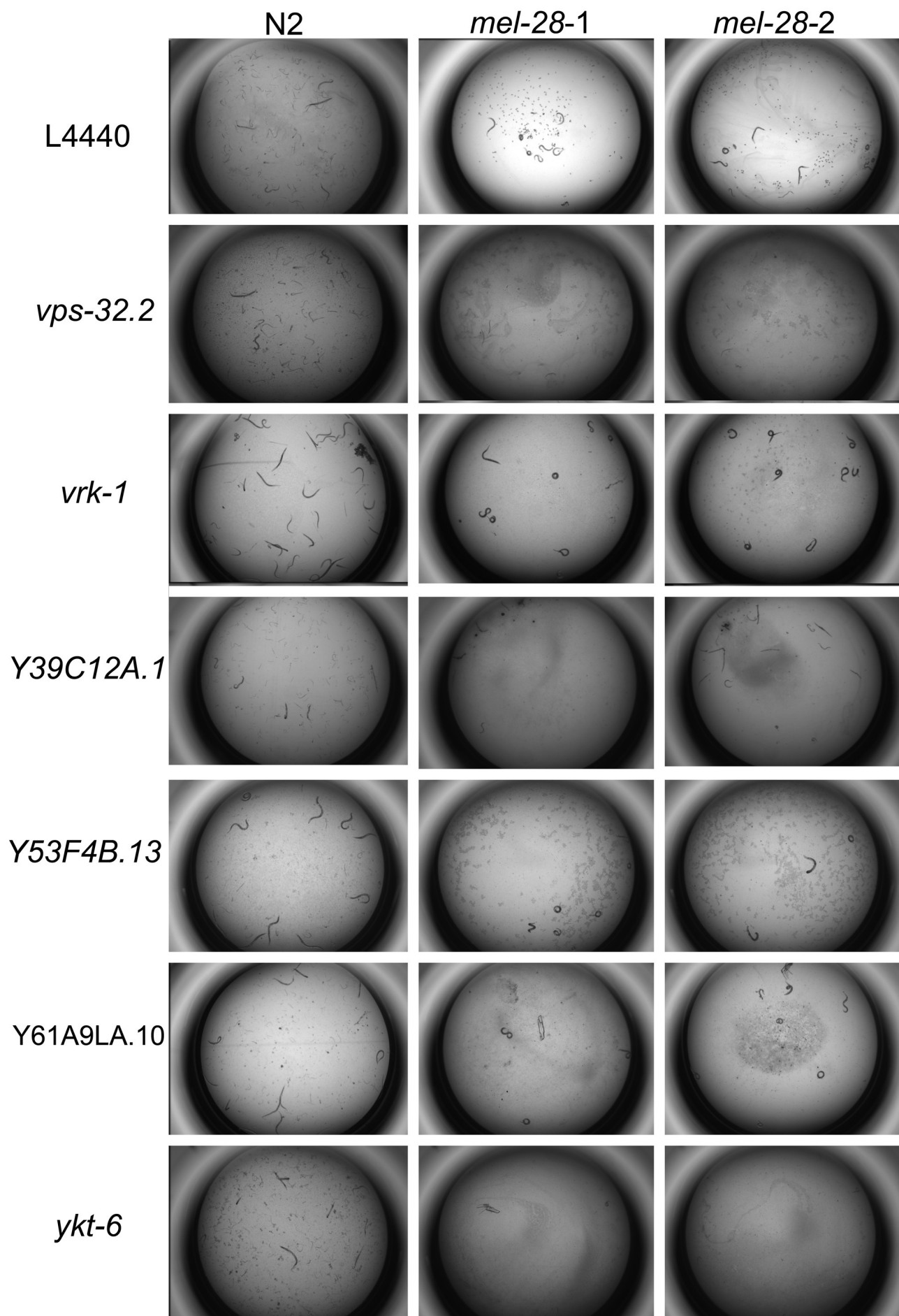

Figure S1 *mel-28* genetic interactor phenotypes

Supplement: Supporting Information [file supp_g3.113.008532_FigureS1.pdf]
