# Supplementary material for: Uncovering Buffered Pleiotropy: A Genome-Scale Screen for mel-28 Genetic Interactors in Caenorhabditis elegans
Source: G3 (Bethesda). 2013 Nov 26;4(1):185–96. doi: 10.1534/g3.113.008532 (PMC3887534; doi:10.1534/g3.113.008532)
Supplement: Supporting Information [file supp_g3.113.008532_TableS1.pdf]

Table S1 Brood size data for *mel-28* genetic interactors

gene	progeny/adult (N2)	eggs/ adult (*mel-28)*	ratio N2 brood size: *mel-28* brood size	genetic interaction
pat-2	25.3	0.8	31.6	synthetic sterility
pat-6	7.3	0.8	9.1	enhancement
klf-3	13.9	0.0	>3	synthetic sterility
his-67	9.9	2.7	3.7	enhancement
his-68	10.2	0.0	>3	enhancement
pyp-1	2.8	0.0	>3	enhancement
ruvb-2	9.5	0.7	13.6	enhancement
dhc-1	1.7	0.0	>3	enhancement
dyci-1	4	0.0	>3	enhancement
dli-1	16	0.4	40	synthetic sterility
dnc-1	14.1	0.6	23.5	synthetic sterility
cap-1	12	0.6	20	enhancement
cap-2	8.4	1.4	6	enhancement
arp-1	16.7	1.8	9.3	synthetic sterility
npp-2	8.7	0.7	12.4	enhancement
npp-4	14.7	0.0	>3	synthetic sterility
npp-5	18.2	1.8	10.1	synthetic sterility
npp-12	21.3	0.3	71	synthetic sterility
npp-14	16.2	0.0	>3	synthetic sterility
npp-15	16	0.0	>3	synthetic sterility
npp-17	12.3	0.3	41	enhancement
npp-20	8.5	0.0	>3	enhancement
npp-22	25	0.0	>3	synthetic sterility
ima-3	6	0.0	>3	enhancement
vrk-1	4	1.1	3.6	enhancement
lpin-1	5	0.0	>3	enhancement
cct-2	17.3	0.2	86.5	synthetic sterility
stc-1	13.6	1.3	10.5	synthetic sterility
sca-1	13.2	0.0	>3	synthetic sterility
phi-56	12.3	0.0	>3	enhancement
K12H4.4	11.8	0.0	>3	enhancement
ggtb-1	2.6	0.0	>3	enhancement
ykt-6	11.7	1.0	11.7	enhancement
syx-4	24.3	0.4	60.8	synthetic sterility
mua-6	18.5	0.7	26.4	synthetic sterility
syd-9	55	0.2	275	synthetic sterility

arf-3	4.2	0.0	>3	enhancement
hgrs-1	17.8	0.0	>3	synthetic sterility
vps-32.2	11.3	0.0	>3	enhancement
Y61A9LA.10	9.4	0.0	>3	enhancement
eif-1	3.7	0.0	>3	enhancement
Y39C12A.1	20.8	0.7	29.7	synthetic sterility
F52C6.2	16	1.8	8.9	synthetic sterility
F52C6.3	14.7	0.0	>3	synthetic sterility
exos-3	11.2	3.0	3.7	enhancement
alg-1	4.9	0.0	>3	enhancement
cyd-1	27.8	0.0	>3	synthetic sterility
ego-2	15	0.6	25	synthetic sterility
dre-1	9.3	0.0	>3	enhancement
nipi-3	8.1	1.0	8.1	enhancement
hmgs-1	15.7	0.0	>3	synthetic sterility
nhr-25	17.1	0.0	>3	synthetic sterility
nhr-67	23.3	0.0	>3	synthetic sterility
apl-1	32	0.0	>3	synthetic sterility
F19B6.1	15.6	0.0	>3	synthetic sterility
C55A6.9	6.8	0.2	34	enhancement
egl-13	25	0.0	>3	synthetic sterility
sox-2	17	0.0	>3	synthetic sterility
gei-13	15	0.4	37.5	synthetic sterility
Y53F4B.13	3.8	0.0	>3	enhancement
lin-3	26.7	0.7	38.1	synthetic sterility
arx-2	27.2	0.0	>3	synthetic sterility
eat-6	10.9	0.0	>3	enhancement
B0250.7	3.8	1.0	3.8	enhancement
sos-1	7.8	0.0	>3	enhancement

For each strain (N2 or *mel-28*) the number of progeny per adult was tallied. We did this by adding up the total number of mothers and dividing this number by the total number of progeny from at least two RNAi experiments. For the *dhc-1* gene these tallies came from a single RNAi experiment (see methods for details).
