# Supplementary material for: Uncovering Buffered Pleiotropy: A Genome-Scale Screen for mel-28 Genetic Interactors in Caenorhabditis elegans
Source: G3 (Bethesda). 2013 Nov 26;4(1):185–96. doi: 10.1534/g3.113.008532 (PMC3887534; doi:10.1534/g3.113.008532)
Supplement: Supporting Information [file supp_g3.113.008532_TableS2.pdf]

Table S2 GO-enriched terms amongst *mel-28* genetic interactors

N	P value	GO attribute ID	GO attribute name
2	2.04E-05	GO:0008290	F-actin capping protein complex
2	6.11E-05	GO:0005787	signal peptidase complex
3	1.06E-05	GO:0005869	dynactin complex
7	2.39E-11	GO:0005643	nuclear pore
3	1.92E-05	GO:0051028	mRNA transport
7	6.97E-11	GO:0046930	pore complex
10	5.02E-13	GO:0005635	nuclear envelope
5	3.34E-06	GO:0006997	nucleus organization
5	2.25E-05	GO:0007338	single fertilization
5	2.73E-05	GO:0009566	fertilization
10	2.10E-08	GO:0031967	organelle envelope
10	2.20E-08	GO:0031975	envelope
12	2.05E-09	GO:0012505	endomembrane system
7	1.07E-05	GO:0051656	establishment of organelle localization
7	1.30E-05	GO:0051640	organelle localization
7	2.82E-05	GO:0032940	secretion by cell
7	3.11E-05	GO:0046903	secretion
8	2.83E-05	GO:0015031	protein transport
11	1.16E-06	GO:0044428	nuclear part
8	3.15E-05	GO:0045184	establishment of protein localization
14	6.34E-08	GO:0051649	establishment of localization in cell
14	1.62E-07	GO:0051641	cellular localization
21	7.91E-10	GO:0043234	protein complex
20	2.37E-09	GO:0006898	receptor-mediated endocytosis
11	8.38E-06	GO:0008104	protein localization
40	9.36E-12	GO:0040007	growth
20	2.91E-08	GO:0006897	endocytosis
21	3.49E-08	GO:0016192	vesicle-mediated transport
42	7.57E-11	GO:0009792	embryo development ending in birth or egg hatching
23	1.75E-08	GO:0044422	organelle part
21	6.33E-08	GO:0044446	intracellular organelle part
21	6.69E-08	GO:0032991	macromolecular complex
42	1.80E-10	GO:0009790	embryo development
33	6.12E-09	GO:0002119	nematode larval development
33	6.24E-09	GO:0002164	larval development
33	7.71E-09	GO:0009791	post-embryonic development

31	1.40E-08	GO:0051234	establishment of localization
30	2.70E-08	GO:0006810	transport
44	3.85E-08	GO:0048856	anatomical structure development
46	7.61E-08	GO:0007275	multicellular organismal development
45	1.52E-07	GO:0044767	single-organism developmental process
24	2.20E-06	GO:0040010	positive regulation of growth rate
24	2.26E-06	GO:0040009	regulation of growth rate
34	2.33E-07	GO:0051179	localization
26	1.56E-06	GO:0040011	locomotion
46	5.04E-07	GO:0032502	developmental process
46	1.46E-06	GO:0044707	single-multicellular organism process
25	6.07E-06	GO:0045927	positive regulation of growth
46	3.83E-06	GO:0032501	multicellular organismal process
19	7.29E-05	GO:0005515	protein binding
25	1.35E-05	GO:0040008	regulation of growth
27	1.21E-05	GO:0048518	positive regulation of biological process
33	5.12E-06	GO:0000003	reproduction
30	5.50E-05	GO:0043229	intracellular organelle
30	5.63E-05	GO:0043226	organelle

We used FuncAssociate (http://llama.mshri.on.ca/funcassociate_client/html/) to determine the GO term enrichment within the list of 65 *mel-28* genetic interactors identified. The background set was the entire *C. elegans* genome.
